# Evaluation of Nitrogen Fertilizer Fates and Related Environmental Risks for Main Cereals in China’s Croplands from 2004 to 2018

**DOI:** 10.3390/plants11192507

**Published:** 2022-09-26

**Authors:** Daping Song, Rong Jiang, Daijia Fan, Guoyuan Zou, Lianfeng Du, Dan Wei, Xuan Guo, Wentian He

**Affiliations:** Institute of Plant Nutrition, Resources and Environment, Beijing Academy of Agriculture and Forestry Sciences, 9 Shuguanghuayuan Middle Road, Haidian District, Beijing 100097, China

**Keywords:** cereal production, nitrogen balance, nitrogen losses, environmental impact, spatial-temporal distribution

## Abstract

Assessment of the nitrogen (N) inputs and outputs in croplands would help effectively manage the distribution of N to improve crop growth and environmental sustainability. To better understand the N flow of the main cereal systems in China, soil N balance, N use efficiency (NUE), N losses and the potential environmental impacts of maize, wheat and rice cropping systems were estimated at the regional and national scales from 2004 to 2018. Nationally, the soil N balance (N inputs—N outputs) of maize, wheat, single rice and double rice decreased by 28.8%,13.3%, 30.8% and 34.1% from 2004–2008 to 2014–2018, equivalent to an average of 33.3 to 23.7 kg N ha^−1^, 82.4 to 71.4 kg N ha^−1^, 93.6 to 64.8 kg N ha^−1^ and 51.8 to 34.1 kg N ha^−1^, respectively. The highest soil N balance were observed in Southeast (SE) region for maize and double rice, North central (NC) region for wheat single rice and Northwest region for wheat, whereas Northeast (NE) region had the lowest N balance for all crops. The NUE increased from 49.8%, 41.2%, 49.7% and 53.7% in 2004–2008 to 54.8%, 45.9%, 55.5% and 56.5% in 2014–2018 for maize, wheat, single rice and double rice, respectively. The fertilizer N losses (i.e., N_2_O emission, NO emission, N_2_ emission, NH_3_ volatilization, N leaching and N runoff) were estimated as 43.7%, 38.3%, 40.2% and 36.6% of the total N inputs for maize, wheat, single rice and double rice, respectively in 2014–2018. Additionally, the highest global warming potential and acidification effects were found in NE and NC regions for maize, NC region for wheat, the middle and lower reaches of Yangtze River for single rice and SE region for double rice, respectively. The highest risk of water contamination by N leaching and surface runoff was observed in NC region for all crops mainly due to high N fertilizer input. Furthermore, the dynamics of N balance for all crops were closely tied with grain yields, except for single rice, the N balance of which was mainly correlated with N fertilizer input. Our results could help researchers and policy makers effectively establish optimized fertilization strategies and adjust the regional allocation of grain cropping areas in response to environmental risks and climate change caused by food crop cultivation in China.

## 1. Introduction

Food security and environmental safety are global concerns affecting humanity [1,2,3], as we will need to produce 60–100% more food by 2050 to feed a world population of 9.6 billion with low environmental risks [4,5]. The increased crop production was mainly driven by the increased fertilizer application, improved crop varieties and optimized agronomic management [6,7]. Over the past decades, the agricultural food production increased by 108.9% along with a 96.3% increase in the use of nitrogen (N) fertilizer in China from 1980 to 2020 [8]. Synthetic N fertilizer application contributed to 30–50% of crop yield, which has significantly improved crop production and reduced yield gaps [9,10]. However, the inappropriate management and imbalanced fertilizer application has not achieved an equivalent increase in yield, resulting in low N use efficiency (NUE) and environmental pollution [11,12]. Water and N are the two most important input factors that affect the growth and development of cereal crops. Hence, optimizing irrigation and N fertilization may help to improve crop production substantially while reducing potential environmental risks [13]. However, it is crucial to balance water and N supply, as an excrescent supply of N could reduce water use efficiency, while excessive water applied could increase N losses through denitrification and leaching [14]. Identifying the NUE and environmental pollution in current intensive crop production is critical to maintain high yields and mitigate environment risks to meet future food requirements.

Globally, maize (*Zea may* L.), rice (*Oryza sativa* L.) and wheat (*Triticum aestivum* L.) are the world’s top three important staple food crops. In China, maize (260.8 Mt), wheat (133.6 Mt) and rice (211.9 Mt) are cultivated in 97.7 M ha, accounting for about 58.7% of the national arable cropland and contributing to 23.5%, 18.0% and 29.8% of the global cereal production in 2019, respectively [15]. Undoubtedly, the demands for N fertilizer in Chinese agriculture will grow substantially for increasing food production to nourish the growing population in the future [16]. Previous reports indicated that N application rate ranged from 45–600, 405–540 and 30–419 kg N ha^−1^ for maize, rice and wheat over the past decades in China, and the partial factor productivity of N ranged from 32–54, 37–48 and 23–38 kg N ha^−1^, respectively [17,18,19]. Compared with China Statistical Yearbook data [8] in early 2000, the partial factor productivity of N has increased by 6.8%, which was mainly attributed to the optimized nutrient management practices based on soil testing, yield responses and 4R (right source, right rate, right time and right place) nutrient stewardship [20]. However, Zhang et al. (2015) reported that the global average NUE in crop production would need to improve from 42% (46%, 42% and 39% for maize, rice and wheat) in 2010 to 68% (70%, 60% and 70% for maize, rice and wheat) in 2050 to meet the dual goals of food security and environmental sustainability [16]. Although numerous field trials have been carried out to investigate the NUE and environmental impacts of the main cereal crops, the lack of a comprehensive assessment of the nutrient balance and environmental risks of different main cereal cropping systems at the regional scale may limit the improvement of the rational nutrient utilization and appropriate distribution of agricultural resources. Therefore, it is critical to estimate the national N balance in crop production to improve the nutrient use efficiency and ultimately achieve food and environmental security.

Global warming, soil acidification, surface water eutrophication and underground water pollution have been reported as results of N losses from agricultural systems [21]. Chemical fertilizer as well as manure from humans and livestock are primary sources of the N-containing air pollutants (e.g., nitrous oxide [N_2_O] and ammonia [NH_3_]) released from agricultural soils into the atmosphere, which then return to the soil through N deposition [22]. Additionally, excessive N fertilizer application led to low NUE and high soil N surplus, which could be further lost to the surface and underground water through nitrate (N) leaching and N runoff, resulting in water eutrophication and underground water contamination [23,24]. Chang et al. (2018) indicated that aquatic eutrophication and soil acidification are significantly relevant to the N fertilizer use in wheat, maize and rice production systems in most of the China Plains [25]. However, the agro-environmental impacts of the N flows and losses in major cereal cropping systems in China have not been comprehensively evaluated at national or regional scales [24,26,27,28,29], which could impede the development of energy-saving and clean production approaches to optimize regional resource allocation, increase crop productivity, improve NUE and reduce environmental impacts of different agro-ecosystems.

Here, we analyzed the historical patterns of N fertilizer application in China’s cereal croplands including maize, wheat and rice (single rice and double rice) fields at the national and regional scales based on the national agriculture statistical database (2004–2018) to determine various environmental pollution pathways induced by N inputs and their driving factors. The objective of this study was to evaluate the soil N balance, NUE and potential environmental risks within the primary cereal production systems at the provincial scales in China during 2004–2018 to support the establishment of a N balance-oriented regional N management strategy.

## 2. Materials and Methods

### 2.1. Data Source and Analysis

To evaluate the N balance in maize, wheat and rice fields at the provincial scales in China, relevant data including chemical fertilizer (simple and compound) N application rate and crop yield for each crop were mainly collected from the China economic development statistics database [30]. Other data including crop sowing areas, population, livestock population and the quantity of surface and underground water in each province were mainly extracted from the China Statistical Yearbook [8]. Two five-year periods of 2004–2008 and 2014–2018 were compared based on the available data. This is due to that the major strategies for increasing Chinese crop production were established in 2004 and updated every five years according to the Chinese National Five-Year-Plan [31]. The missing values were interpolated using the data from adjacent years, referring to other statistical yearbooks, published reports and papers.

Statistical analysis was performed using SPSS (SPSS Inc., CHI, IL, USA; Version 21.0). The normality and homogeneity of variance were verified beforehand using Shapiro-Wilk and Levene tests to ensure that all data meet the requirements for analysis of variance (ANOVA). One-way ANOVA was conducted to analyze the data, followed by Tukey honest significant difference test when the treatment effects were significant. All levels of significance were defined at *p* ≤ 0.05. The maps of spatial distribution in different regions were generated using ArcGIS (Esri Corporation, RE, CA, USA; Version 10.8).

**Figure 1:**
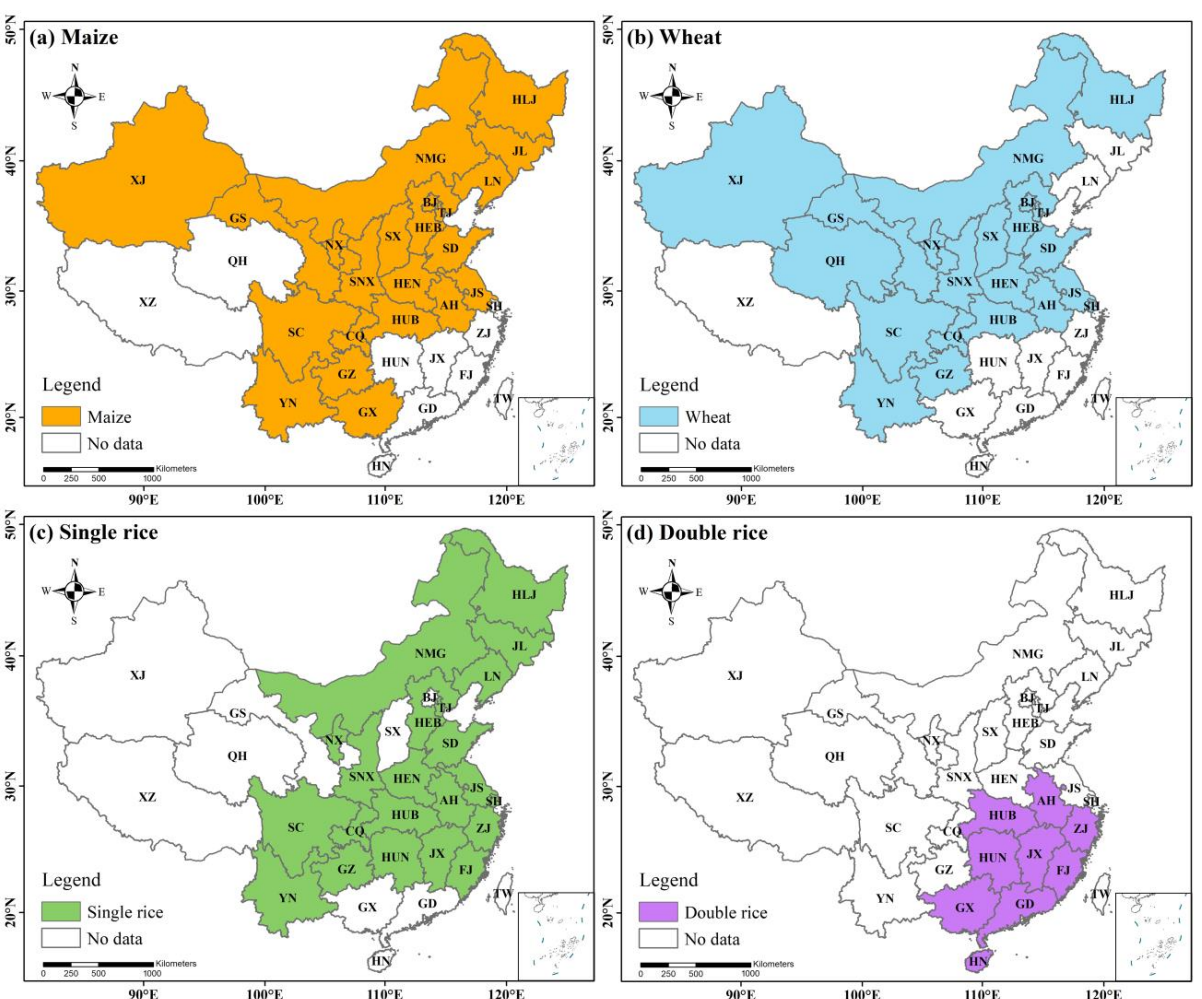
The distribution of field for (**a**) maize, (**b**) wheat, (**c**) single rice and (**d**) double rice systems from 2004 to 2018 in China. The four coloured regions represent different cultivated zones for (**a**) maize, (**b**) wheat, (**c**) single rice and (**d**) double rice.

The agricultural regions were categorized into six groups based on geographical locations and China’s administrative divisions (Table S1), including northeast (NE), north central (NC), northwest (NW), the middle and lower reaches of the Yangtze River (MLYR), southeast southwest (SE) and southwest (SW) regions, to evaluate the regional variations in N fertilizer fates and N-related environmental risks (i.e., N_2_O emission, NO emission, N_2_ emission, NH_3_ volatilization, N leaching and N runoff). Note that Qinghai and Tibet were not included in this study because cereals are rarely grown in these regions due to climatic limitations. Maize planting areas are mainly distributed in the NE, NC, NW and SW regions. Wheat planting areas are mainly distributed in the NC, MLYR and SW regions. Single rice is mainly planted in the NE, MLYR and SW regions while double rice is only planted in the MLYR and SE regions. The main planting area for each crop accounts for more than 90% of the national planting area (Figure 1).

### 2.2. Calculation of N Balance

Nitrogen balance indicates the difference between the total N input into the soil and the total N output for each hectare of cropland [32] and the equations are shown as follows:

(1) $$N_{input}=N_{fert}+N_{man}+N_{straw}+N_{fix}+N_{depo}+N_{irri}$$

(2) $$N_{\mathrm{output}}=N_{\mathrm{crop}\_\mathrm{removal}}+N_{N2O}+N_{\mathrm{NH}3}+N_{\mathrm{NO}}+N_{N2}\;+N_{N-\mathrm{leaching}}+N_{N-\mathrm{runoff}}$$

(3) $$N_{balance}=N_{input}–N_{output}$$

where, N*_input_* (kg N ha^−1^) is the sum of chemical fertilizer N (N*_fert_*), manure fertilizer N from livestock and humans (N*_man_*), straw N returned to the soil (N*_straw_*), N fixed via symbiotic and non-symbiotic pathways (N*_fix_*), dry and wet N deposition from the atmosphere (N*_depo_*) and N from irrigation water (N*_irri_*). The N*_input_* parameters based on previous studies are listed in Tables S2–S4 in the Supplementary Materials. The N*_output_* (kg N ha^−1^) is the sum of N removal (N*_straw_* + N*_grain yield_*) from crops, grain yield N (N*_grain yield_*), N_2_O emissions (N*_N2O_*), NH_3_ volatilization (N*_NH3_*), NO emissions (N*_NO_*), N_2_ emissions (N*_N2_*), N leaching (N*_N-leaching_*) and N (the sum of ammonium NH_4_^+^-N and NO_3_^−^-N) runoff (N*_N-runoff_*). The N*_output_* parameters based on previous studies are shown in Table S5.

If $N_{balance}$ = 0, there is no N surplus in the soil in terms of annual budget. If $N_{balance}$ < 0, it indicates a potential N stress in the soil and the necessity for increasing N inputs to meet crop demand. If $N_{balance}$ > 0, it indicates a potential N surplus in the soil and the need for optimizing N management to increase NUE and reduce N losses.

### 2.3. Assessment of NUE

The NUE is defined as the ratio of N contained in agricultural crop products to N input [18,33], and the equation is shown as follows:

(4) $$\mathrm{NUE}\;={\;N}_{\mathrm{crop}\_\mathrm{removal}}\;/{\;N}_{\mathrm{input}}\times 100\%$$

### 2.4. Estimation of Fertilizer N Losses

Fertilizer N loss ratio (NLR) indicates the environmental fate of N fertilizers including the N losses through gaseous emissions (N_2_O, NH_3_, NO and N_2_), and N surface runoff and N leaching [24]. The equation is shown as follows:

(5) $$\mathrm{NLR}\;=\;\mathrm{TNL}\;/{\;N}_{\mathrm{input}}\times 100\%$$

where, TNE is the total N loss (kg N ha^−1^).

#### 2.4.1. Global Warming Potential

Global warming potential (GWP) is used to evaluate the environmental impacts of greenhouse gas (i.e., N_2_O) and is defined as the relative radiation effect of a given substance compared to that of carbon dioxide over a period. The equation is shown as follows:

(6) $$\mathrm{GWP}=N_{N2O}\times n$$

where, *n* is the global warming equivalent coefficient of N_2_O (265) [34,35].

#### 2.4.2. Acidification Potential

Acidification potential (AP) is used to evaluate the environmental impacts of NH_3_, which is calculated according to the equation below:

(7) $$\mathrm{AP}=N_{NH3}\times nh$$

where, *nh* is the acidizing equivalent coefficient of NH_3_ (1.88) [24,36].

#### 2.4.3. Water Pollution Induced by N Surface Runoff and Leaching

Water pollution level (WPL), which measures the degree of pollution within water bodies, is estimated as the ratio of total gray water footprint (GWF) to actual water resources, including surface water and underground water [24,37]. The GWF is the amount of freshwater needed to assimilate pollutant load based on natural background and maximum allowable concentrations of that pollutant [37]. The equation is shown as follows:

(8) GWF = L / (Cmax−Cnat)

(9) WPL = GWF / WR

where, L is the N losses via surface runoff or leaching (t N yr^−1^), C*_max_* and C*_nat_* are the maximum allowable and natural concentrations of N in surface water or underground water, respectively (g N m^−3^), WPL is the water pollution level of surface or underground water, and WR is the actual water resources of surface water or underground water (m^3^ yr^−1^). According to Sun et al. (2020), C*_max_* is 2.0 mg L^−1^ for surface water, and C*_max_* is 31.5 mg L^−1^ for underground water (the sum of ammonium NH_4_^+^-N and NO_3_^−^-N) [24]. The natural background concentration is obtained by natural and geological processes without anthropogenic contribution. The GWF guideline from the Water Footprint Network suggests a natural total N concentration of 0.4 mg N L^−1^ in surface water [24]. For underground water, a natural total N concentration of 1.2 mg L^−1^ was determined as the median value of previous results in China. The parameters of N losses via N surface runoff and leaching are described in Table S5.

## 3. Results and Discussion

### 3.1. Spatial and Temporal Variations in N Balance

**Figure 2:**
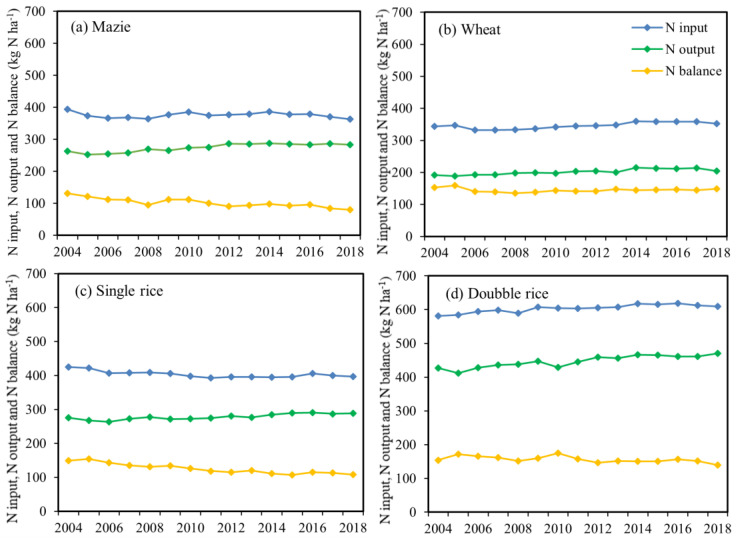
Nitrogen (N) input, N output and N balance per hectare (kg N ha^−1^) from 2004 to 2018 for (**a**) maize, (**b**) wheat, (**c**) single rice, (**d**) double rice at national scale in China’s farmlands, respectively.

The spatial and temporal variations in N balance exhibited surplus during 2004–2018 for all crops at both national and regional scales (Table 1 and Figure 2). The high N balance was mainly attributed to high N input and low crop N removal. At the national scale, the N balance of maize, wheat, single rice and double rice was 23.7, 71.4, 64.8 and 34.6 kg N ha^−1^ in the 2014–2018, respectively, which was 28.8%,13.3%, 30.8% and 34.1% lower than in 2004–2008 (Table 1). For all crops, wheat had the highest N surplus, followed by single rice and double rice, and maize. Additionally, the N balance of maize and single rice showed a downward trend after 2014, which was mainly due to that the increase in crop N removal was higher than that of N input (Figure 2). There was no significant difference in N balance between the two periods for wheat and double rice because of the stable crop N removal and N input (Figure 2, Tables S6 and S7). The soil N balance in this study was lower than previous results [38,39,40], which were 165, 138.32 and 133.4 kg N ha^−1^ for wheat, maize and single rice from 2005 to 2014 in China, respectively. This is due to that the N losses via N_2_O emission, NO emission, N_2_ emission, NH_3_ volatilization, N surface runoff and N leaching were not included in previous studies. Compared to other countries, the N balances for maize and single rice in our study were lower than Japan, Korea and the Netherlands (above 150 kg N ha^−1^) [41]. And the N balances for maize were lower than USA and Canada (88 kg N ha^−1^ and 80 kg N ha^−1^ for maize in 2010s) due to a higher crop removal and a lower amount of livestock manure input [42]. This implies great environmental impacts of the cereal cropping systems in China, given that the risk and magnitude of N losses would increase substantially when the N balance is above 75 kg N ha^−1^ [43,44,45].

The lowest N balance for all crops occurred in the NE region, especially in HLJ province, mainly due to the higher N output by crop yield and the lower N input (Table 1, Table S6). The decreasing trend of N balance was in accordance with the findings of He et al. (2018) and Wang et al. (2016), who reported that the N balance in China’s croplands decreased slightly from 2004 to 2014 [18,46]. Meanwhile, Wang et al. (2014) observed soil N surplus in most counties in China’s croplands [28].

**Figure 3:**
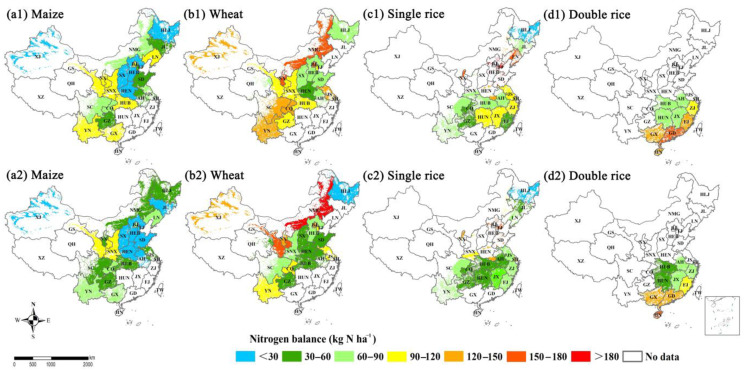
Nitrogen balance (NB) per hectare (kg N ha^−1^) after harvest between 2004–2008 and 2014–2018 for (**a1**,**a2**) maize, (**b1**,**b2**) wheat, (**c1**,**c2**) single rice and (**d1**,**d2**) double rice at provincial scale in China’s farmlands, respectively.

For maize, the hotspots with higher levels of N balance were observed in Gansu (GS), Shaanxi (SNX) and Ningxia (NX) provinces of NW region, Guangxi (GX) and Yunnan (YN) provinces of SW region, and Liaoning (LN) province of NE region in 2004–2008 (Figure 3(a1)), while its N balance declined to a moderate level in the NE and NC regions in 2014–2018 (Figure 3(a2)). The hotspots with higher levels of N balance for wheat covered a larger area of NW region (i.e., NMG, XJ and NX) and SW region (i.e., SC, CQ and YN) in 2004–2008 than that in 2014–2018 (Figure 3(b1,b2)). Meanwhile, the hotspots with higher levels of N balance for single rice were mostly distributed in NC region (i.e., BJ, TJ, HEB, HEN and SD) and MLYR region (i.e., JS, SH and ZJ) in 2004–2008 (Figure 3(b1,b2))., but it decreased to low-to-moderate levels and expanded to more areas in 2014–2018 (Figure 3(c1,c2)) such as most provinces in NE (i.e., HLJ and JL), MLYR (i.e., HUB, HUN, ZJ, JX and AH) and SW (i.e., CQ, SC, GZ and YN) regions. For double rice, the areas with high N balance mostly distributed in SE (i.e., GUX, HN, GD and FJ) regions in China between 2004–2008 and 2014–2018 (Figure 3(d1,d2)). The annual seeding area of maize and wheat in China from 2004 to 2018 increased by 66.7% and 12.8%, respectively (Figure S1). There was no significant difference for rice planting area between 2004 and 2018, but the planting area of single rice increased and that of double rice decreased during that time under the influence of national policies and market demand [47,48]. Overall, the variations in the amount of fertilizer input in different regions and the nutrient requirements of different crops led to the significant spatial variations in the N balance of the same crop among different regions or different crops in the same region. For example, the soil N balance of maize in west China was significantly higher than that in east China. Besides, this is closely correlated with the large spatial variations in temperature, precipitation, soil texture, soil organic matter, land use and management practices across China, all of which exert great influence on soil N pools, N cycling and N balance [44]. Our regional and provincial scale results indicated that high resolution analysis could help explore the areas with high N balance as well as the correlations of the N balance among different crops in more details.

The relationship between N balance and fertilizer N input indicated that the dynamics of N balance was mainly regulated by fertilizer N input. The N inputs from chemical fertilizer and manure were the main driving factor affecting the N balance for all crops. Increased N fertilizer inputs in croplands could guarantee crop yields, whereas excessive fertilizer application could induce an imbalance between N input and output and result in environmental pollution and nutrient loss [49,50]. Nitrogen fertilizer application showed a significant positive correlation with N uptake, soil inorganic N content and crop N removal. Taking the factors including crop yields, NUE and soil inorganic N accumulation all into consideration, the optimum N application rate ranged from 180 to 220 kg N ha^−1^ in the main crop production regions of China [38,39]. In China, a large amount of N fertilizer was applied to cropland by small-holder farmers in order to increase crop yield, especially in high-intensity agricultural areas [48]. Nitrogen balance for a field is a measure of the extent to which anthropogenic N supply exceeds crop needs. Although modest excess may be required (e.g., to support growth of unharvested plant parts and maintain soil organic matter), a large excess creates a pool of reactive N in soil that is extremely vulnerable to loss and is therefore a potential source of pollution [44]. Thus, it is important to determine if certain management practices can effectively reduce N balance by increasing crop yield (e.g., optimizing plant density, cultivar maturity and sowing date) or reducing N inputs (e.g., 4R fertilizer management).

### 3.2. Integrated Analysis of the Fate of N Fertilizers

**Figure 4:**
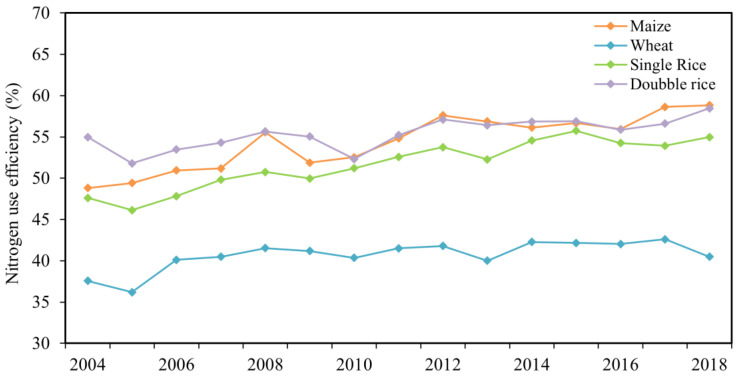
Nitrogen use efficiency (%) for maize, wheat, single rice and double rice from 2004 to 2018 in China farmlands, respectively.

The NUE showed a fluctuating upward trend for all crops from 2004 to 2018 (Figure 4) and increased from 49.8% to 54.8%, 41.2% to 45.9%, 49.7% to 55.5% and 53.7% to 56.5% for maize, wheat, single rice and double rice, respectively (Table 2). The increased NUE was mainly owing to higher crop yield and lower N input. The highest NUE was from maize cropping system, followed by double rice and single rice, with the lowest NUE observed for wheat. The increasing trend of NUE was consistent with previous study claiming that the NUE in China’s croplands increased by 6.8% from 2004 to 2014 [51]. The average NUE for maize and wheat during 2004–2018 in this study was in accordance with the previous reported results for China’s cropping systems (i.e., 46% for maize, 42% for wheat and 39% for rice) [19]. Some studies reported that the N application rates in China were substantially higher and the NUE was much lower than developed countries such as North America, Europe and Australia, the NUE of which ranged from 68% to 75% according to the statistical data from FAO [16,42]. The increase in NUE and the reduction in soil N surplus in most croplands are owing to modern agricultural management practices, such as reduced inorganic N inputs [52,53], application of controlled-release N fertilizer [54], combined application of organic and inorganic fertilizers and optimized tillage practices [36,55]. However, more than 30% of the agricultural N inputs could lead to potential environmental hazards. In China’s croplands, NUE had increased greatly after 2004, but a big gap still exists compared with developed countries [15]. Previous field experiments indicated that the average NUE increased by 10–20% under different cropping systems, such as the winter wheat-summer maize-spring maize rotations in the North China plain and the maize-soybean intercropping in southeast China [56]. It was mainly due to the adoption of improved agricultural management practices such as reduced use of fertilizers and pesticides, water-saving irrigation, and crop intercropping and rotation.

### 3.3. Environmental Impacts of N Losses

The increased N inputs without appropriate fertilizer management decreased NUE, resulting in higher nutrient losses to the soil, water and air. Total N losses via N_2_O emission, NO emission, N_2_ emission, NH_3_ volatilization, N surface runoff and N leaching for wheat and double rice slightly increased by 12.1% and 5.4% in 2014–2018 compared to that in 2004–2008, respectively, while it decreased by 2.0% and 4.3% for maize and single rice (Table 3). The increased N loss from 2004 to 2014 was partially due to the increased fertilizer N input. In contrast, the reduction in N losses through different pathways during 2014–2018 was largely owing to the Chinese government’s efforts to discourage pollution, reduce fertilizer input and increase NUE in the past decades [55]. The highest N loss occurred in NC region for maize and wheat, with different temporal trends for each region. Double rice had the highest N loss (231.6 kg N ha^−1^), followed by maize (162.4 kg N ha^−1^), wheat (140.3 kg N ha^−1^) and single rice (120.4 kg N ha^−1^) in the 2014–2018 period. Over the 15-year period, NH_3_ volatilization, N_2_ emission and N leaching were the major contributors (accounting for more than 85%) to the total N loss for all crops (Figure S4). This is mainly attributed to the widespread use of chemical fertilizers including urea, ammonium sulfate and ammonium bicarbonate across China [17,24]. The N loss factors varied across regions and crops with different climatic conditions, topography, agricultural management practices (e.g., tillage, irrigation and rotation system) and soil properties. For rice, the highest N loss was through NH_3_ volatilization, followed by N_2_ emission, N leaching, N surface runoff and N_2_O emission, and the lowest N loss was via NO emission (Figure S4). For maize and wheat, the highest N loss was through N leaching, followed by N_2_ emission, NH_3_ volatilization, N surface runoff and N_2_O emissions, and the lowest N loss was via NO emission (Figure S4). The variations in both N balance and precipitation led to the significant changes in N leaching and N surface runoff in crop growing seasons. Meanwhile, the ratio of N loss as surface runoff in north China was lower than that in south China, whereas the ratio of N loss via leaching was higher than south China. This is because the rainfall and surface water resources in north China are significantly lower than south China [24,50]. Thus, the water pollution caused by N losses in maize and wheat cropping systems in north China is dominated by leaching, while surface N runoff from the rice cropping systems in south China is the main contributor [56,57,58].

**Figure 5:**
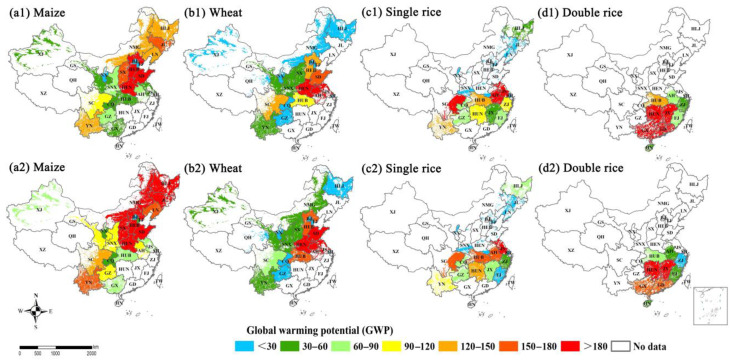
The global warming potential (GWP) between 2004–2008 and 2014–2018 for (**a1**,**a2**) maize, (**b1**,**b2**) wheat, (**c1**,**c2**) single rice and (**d1**,**d2**) double rice at provincial scale in China’s farmlands, respectively.

**Figure 6:**
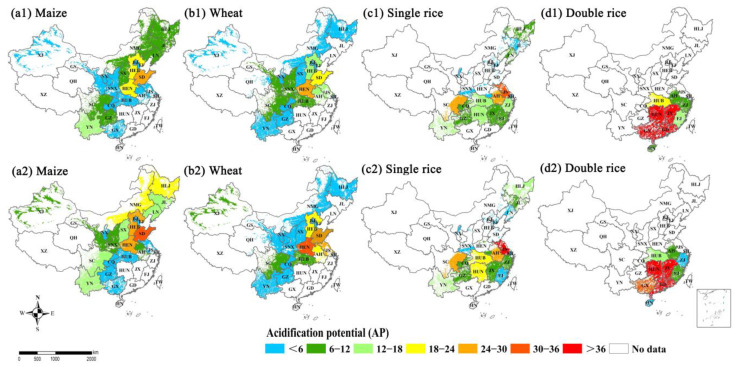
The acidification potential (AP) between 2004–2008 and 2014–2018 for (**a1**,**a2**) maize, (**b1**,**b2**) wheat, (**c1**,**c2**) single rice and (**d1**,**d2**) double rice at provincial scale in China’s farmlands, respectively.

The N loss-induced environmental impacts were compared at regional and provincial scales, and they varied spatially across China’s croplands (Tables S8–S11). The areas of hotspots with high global warming potential (GWP) increased for maize and wheat, while they decreased for single rice and double rice during the 2004–2008 and 2014–2018 periods (Figure 5). The hotspot areas for maize included NE (HLJ, JL, and LN provinces) and NC regions (HEB, HEN, SX and SD provinces) and for wheat NC region (HEB, HEN, and SD provinces) and MLYR (AH, JS, and HUB provinces) region. For single rice and double rice, the hotspot areas were in the MLYR (AH, HUB, HUN, JS and JX provinces) and SE (GD and GX provinces) regions. The hotspot areas with high acidification potential (AP) basically overlapped with those with high GWP, which included the NC (HEB, HEN and SD provinces) region for maize, NC (HEN and SD provinces) region for wheat, MLYR (JS, AH, HUB and HUN provinces) region for single rice and SE region (GD and GX provinces) for double rice. In addition, the hotspot area for maize was observed to increase in NMG of Northwest (NW) China. The spatial and temporal variations in the N loss-induced environmental risks were mainly associated with the increased N input from chemical fertilizer and manure (Figure 6).

**Figure 7:**
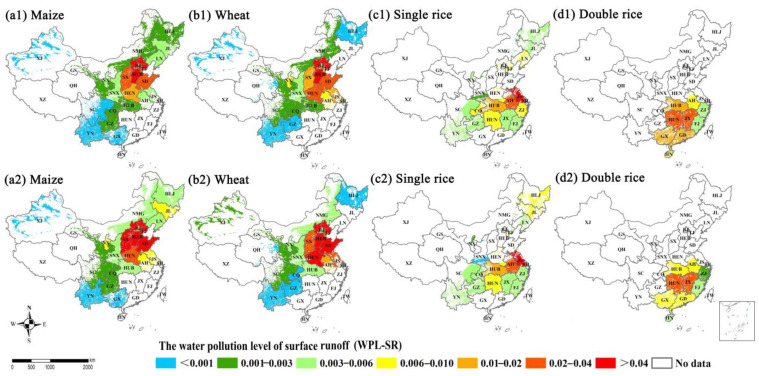
The water pollution level of surface runoff losses (WPL-SR) between 2004–2008 and 2014–2018 for (**a1**,**a2**) maize, (**b1**,**b2**) wheat, (**c1**,**c2**) single rice and (**d1**,**d2**) double rice at provincial scale in China’s farmlands, respectively.

**Figure 8:**
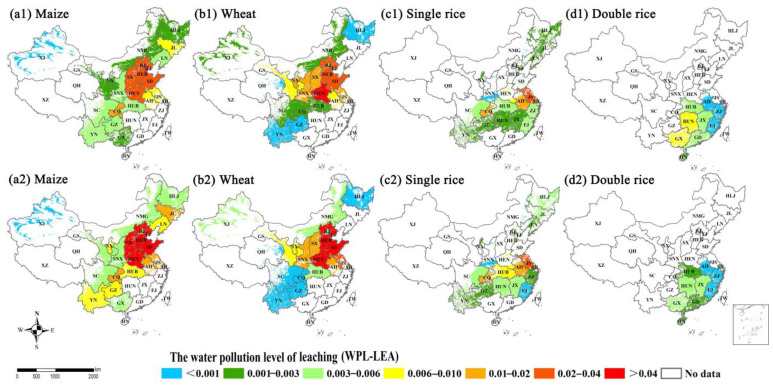
The water pollution level of leaching losses (WPL-LEA) between 2004–2008 and 2014–2018 for (**a1**,**a2**) maize, (**b1**,**b2**) wheat, (**c1**,**c2**) single rice and (**d1**,**d2**) double rice at provincial scale in China’s farmlands, respectively.

In this study, the highest water pollution level (WPL) caused by N surface runoff and leaching were found in the NC region, which could be partly due to the excessive fertilizer input, N losses and shortage of water resources. Similarly, previous findings of Sun et al. (2020) and Wang et al. (2018) showed that the regions with high water pollution risk posed by agricultural N loss were distributed in north China [24,59]. Meanwhile, part of NE (i.e., LN, and JL provinces), NC (i.e., HEB, HEN and SD provinces) and MLYR (i.e., HUB, JS and AH provinces) had also been identified as the hotspots of water pollution for maize, wheat and single rice. Water pollution risk was relatively low in the SE and MLYR regions where double rice was grown (Figure 7(d1,d2) and Figure 8(d1,d2)), which was because the SE and MLYR regions are richer in surface water and underground water resources than other regions. A more detailed analysis of the spatial distribution of N loss-induced water pollution is required to reveal the hotspots of gaseous N loss due to flooded rice cultivation and ample rainfall [60]. The hotspot areas with high N discharge to water bodies have expanded from 2004–2014 to 2014–2018 for maize and single rice, indicating a greater risk of N loss-induced water pollution.

This study showed that fertilizer N management played an important role in mitigating agricultural N losses. Drury et al. (2017) reported that urea amended with urease inhibitor reduced the NH_3_ volatilization by 60% but increased the N_2_O emissions by 30% [61]. Additionally, some other studies indicated that optimized N fertilizer application timing and fertilizer deep-band placement could simultaneously increase crop N uptake, NUE and maize yield, and decrease soil N surplus, N losses and GHG emissions [62,63]. Cui et al. (2018) reported that Chinese government encouraged 20.9 million smallholders to adopt enhanced fertilizer management techniques to obtain high yield and reduce environmental pollution, though the number of participants is still rather limited [50]. Thus, the adaptability of the optimized fertilizer management practices under different climatic and soil conditions should be further analyzed in future research. Besides, it is important to consider establishing climate-smart N management strategies to determine the corresponding N fertilizer application method under various climatic conditions [64]. Overall, more attention should be paid to the development of sustainable agriculture that could balance grain yield and environmental conservation. Optimizing N fertilizer management and developing water-saving agricultural approaches could efficiently prevent N losses and water pollution. Conservation tillage combined with straw return could realize the sustainable development of agriculture by improving soil quality and reducing non-point source N pollution [65]. In addition, developing climate-resilient crop varieties and region-specific crop rotation systems could also maintain high crop yield while reducing environmental risks [66].

### 3.4. Correlations among N Balance, Crop Yield and N Fertilizer Input

**Figure 9:**
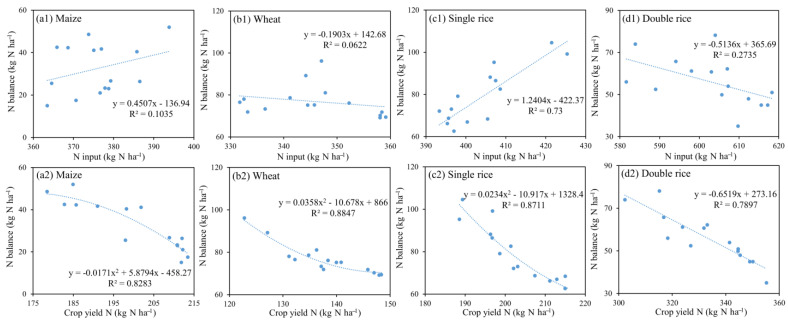
The relationship between N balance and N input from chemical fertilizer and manure for (**a1**) Maize, (**b1**) Wheat, (**c1**) Single rice and (**d1**) Double rice and relationship between N balance and crop yield N for (**a2**) Maize, (**b2**) Wheat, (**c2**) Single rice and (**d2**) Double rice during the period of 2004–2018 across China’s farmlands, respectively.

The N balance was shown to be positively correlated with N fertilizer input with relatively low R^2^ values for maize (R^2^ = 0.10) from 2004 to 2018 (Figure 9(a1)), implying that other factors (e.g., climate, irrigation and tillage) could be the main drivers affecting the N balance in soil [63]. There was a significant positive correlation between N balance and N fertilizer input rate for single rice (R^2^ = 0.73) (Figure 9(c1)), indicating that excessive N fertilizer input was the major cause of N surplus in soil. In contrast, N balance was negatively correlated with N fertilizer input rate for wheat and double rice (Figure 9(b1,d1)), showing that the dynamics of N balance was mainly demand-driven by the nutrients required for the growth of double rice. The N balance was negatively correlated with crop N removal for maize (R^2^ = 0.83), wheat (R^2^ = 0.88), single rice (R^2^ = 0.87) and double rice (R^2^ = 0.79) (Figure 9(a2–d2)), which indicated that low crop removal of N was a main contributor to the N surplus in soil. Numerous field studies have demonstrated that crop yields generally increase with N fertilizer input until a threshold is reached, but excessive N fertilizer input would lead to a decrease in NUE and increased N losses rather than further stimulating crop yields [67,68]. In general, the significant variation in the spatio-temporal distributions of different cropping systems should be taken into consideration to balance the regional agricultural development and environmental preservation across China [69].

## 4. Uncertainties and Limitations

There still exist uncertainties and limitations in the comprehensive assessment of the N input and output of cereal cropping systems at the regional and provincial scales across China in this study, mainly due to the variations in the pathways of N input and corresponding N loss factors. In this study, two datasets from the China Agricultural Products Cost-benefit Compilation of Information and China Statistical Yearbook were used to ensure the comprehensiveness of the results. Though the recognized authority of these data sources guarantees great consistency and ensures comparability of the obtained results [24], they may still lead to variations and uncertainties in the final results given their different ways of data collection. Besides, our results did not reveal the differences in the impact of field management practices on crop yield and N losses in different regions due to data limitation. For example, the application amount of organic fertilizer was determined according to local farmers’ practices, which varied with regions and crops. Also, the regional parameters used in N loss assessment may cause some uncertainty in the results. In addition, current datasets often fail to reflect the different farmers’ practices at regional and provincial scales in detail, such as N fertilizer management (i.e., rate, placement and timing), tillage (i.e., no-tillage and rotary tillage) and irrigation (i.e., rainfed, flooded and drip irrigation), which could largely affect the N balance and N losses [70,71]. Moreover, uncertainty could also arise from the use of natural N input and output factors. In this study, previously reported natural N input factors in different regions and cropping systems in China were collected as reference. Thus, local N input and output models were established based on previous field experiments and water quality studies to estimate the direct and indirect N input and output in different regions and croplands [18,24,72,73]. In addition, the areas where cereal crops are widely studied were selected for our analysis to ensure the availability of sufficient data and the robustness of the final results for each crop at different scales across China. Some of the previous studies used the default emission factors from the IPCC (2013) to estimate the direct GHG emissions at the agricultural materials stage and arable farming stage. While IPCC emission factors are suitable for global assessment, they need further validation and updates for regional studies [71]. The aforementioned factors and their interactions made it difficult to propose generalized or universal strategies. This study provided insights for a more comprehensive understanding of the N flows under different agricultural management practices and identified the hotspot areas with high N balance and N losses in the major cereal cropping systems at regional and provincial scales in China, and ultimately facilitated the establishment of the optimal agricultural management strategies to reduce the N loss-induced environmental risks and to improve NUE and crop production.

## 5. Conclusions

The temporal and spatial patterns of N flows and related environmental impacts for the major cereal crops in China were evaluated from 2004 to 2018. Significant spatial variations in N balance, NUE and N losses were observed for all cereal crops, which were mainly attributed to the large regional differences in N fertilizer input and cultivation methods. Overall, the N balance decreased from 2004–2008 to 2014–2018 for most regions where maize, wheat and rice were grown mainly due to the increased NUE via the adoption of modern agricultural management practices, such as reduced fertilization, application of controlled-release N fertilizer, combined application of organic and chemical fertilizers, reduced tillage and improved crop varieties.

The N leaching was the dominant N loss pathway compared with surface runoff for maize, wheat and rice in the most regions of China. Thus, the environmental impacts caused by N surface runoff and leaching presented different spatio-temporal pattern for different crops. The variations in AP basically followed the same pattern with that in GWP in most areas. The hotspot areas occurred in NC and NE regions for maize and wheat and in MLYR and SE regions for single and double rice. The highest WPL-SR and WPL-LEA occurred in NC, MLYR and SE regions of China.

This study could help the policy makers identify the areas with potentially high risks of N accumulation and N loss, and then establish agriculture adaptation strategies to address the agronomic and environmental problems. However, the results of this study, especially those in high-level environmental risk areas, should be further validated by field experiments to determine the upper limit of N fertilizer application rates and optimize the N management practices for different cereal crops across China. In addition, the socio-economic benefits should be taken into account together with environmental indicators in future research to avoid potential economic loss in regional agricultural production.

## Figures and Tables

**Table 1:** Nitrogen (N) balance (kg N ha^−1^) for maize, wheat, single rice and double rice between the 2004–2008 and 2014–2018 at the region and national scale in China’s farmlands, respectively.

Region ^1^	Maize		Wheat		Single Rice		Double Rice	
	2004–2008	2014–2018	2004–2008	2014–2018	2004–2008	2014–2018	2004–2008	2014–2018
NE	43.1 ± 21.2 b ^2^	31.5 ± 15.3 b	47.5 ± 14.9 b	17.2 ± 6.3 c	87.8 ± 41.2 c	47.9 ± 25.2 e	-^3^	-
NW	80.2 ± 31.4 a	60.8 ± 26.2 a	120.8 ± 43.9 a	125.5 ± 51.2 a	133.8 ± 51.5 b	117.4 ± 34.3 b	-	-
NC	26.2 ± 22.9 c	3.1 ± 15.0 c	77.8 ± 36.5 a	49.7 ± 25.2 b	201.5 ± 44.3 a	147.3 ± 18.2 a	-	-
MLYR	48.8 ± 17.3 ab	18.3 ± 10.8 b	44.8 ± 21.6 a	34.0 ± 15.9 ab	57.5 ± 16.5 c	34.8 ± 10.7 c	23.2 ± 6.5	10.4 ± 3.3
SE	68.1 ± 9.6 a	68.2 ± 10.6 a	-	-	39.2 ± 7.2 c	29.9 ± 5.6 cd	80.5 ± 13.8	78.8 ± 10.2
SW	47.8 ± 11.7 ab	24.6 ± 8.4 b	78.0 ± 16.5 a	73.7 ± 19.5 ab	36.8 ± 6.5 c	16.5 ± 4.7 cd	-	-
China	33.3 ± 26.1	23.7 ± 25.4	82.4 ± 40.1	71.4 ± 35.4	93.6 ± 33.8	64.8 ± 26.9	51.8 ± 10.3	34.1 ± 10.2

^1^ NC, north central; NW, northwest; MLYR, the middle and lower reaches of the Yangtze River; SE, southeast; SW, southwest. ^2^ Same letter within column means no significant difference between treatments at the level of 0.05. ^3^ “-” indicates that the crop is not planted in the area.

**Table 2:** Nitrogen use efficiency (NUE, %) for maize, wheat, single rice and double rice between the 2004–2008 and 2014–2018 at the region and national scale in China’s farmlands, respectively.

Region ^1^	Maize		Wheat		Single Rice		Double Rice	
	2004–2008	2014–2018	2004–2008	2014–2018	2004–2008	2014–2018	2004–2008	2014–2018
NE	66.7 ± 12.9 a ^2^	69.9 ± 13.3 a	52.9 ± 7.9 a	68.3 ± 2.9 a	58.4 ± 13.1 a	66.9 ± 8.1 a	-^3^	-
NW	58.0 ± 10.4 b	63.4 ± 8.7 ab	40.5 ± 6.7 b	42.0 ± 8.2 b	49.0 ± 7.7 bc	52.7 ± 6.2 cd	-	-
NC	48.3 ± 8.8 c	57.0 ± 9.7 bc	38.4 ± 7.2 b	41.4 ± 7.9 b	40.5 ± 4.0 d	48.9 ± 2.3 d	-	-
MLYR	42.7 ± 5.0 cd	49.1 ± 5.0 d	42.4 ± 7.5 b	43.6 ± 7.2 b	46.6 ± 5.7 c	51.8 ± 5.3 cd	56.4 ± 3.6	60.7 ± 3.6
SE	38.1 ± 1.9 d	37.9 ± 2.7 e	-	-	52.8 ± 2.2 b	55.2 ± 1.7 bc	51.1 ± 3.1	52.2 ± 4.5
SW	45.0 ± 6.3 cd	51.4 ± 4.3 cd	31.6 ± 2.7 c	34.2 ± 3.6 c	50.8 ± 4.9 bc	57.7 ± 3.6 b	-	-
China	49.8 ± 12.1	54.8 ± 11.6	41.2 ± 7.9	45.9 ± 9.7	48.5 ± 8.7	54.7 ± 7.6	54.0 ± 4.3	56.9 ± 5.8

^1^ NC, north central; NW, northwest; MLYR, the middle and lower reaches of the Yangtze River; SE, southeast; SW, southwest. ^2^ Same letter within column means no significant difference between treatments at the level of 0.05. ^3^ “-” indicates that the crop is not planted in the area.

**Table 3:** Nitrogen losses of fertilizer (kg N ha^−1^) for maize, wheat, single rice and double rice between the 2004–2008 and 2014–2018 at the region and national scale in China’s farmlands, respectively.

Region ^1^	Maize		Wheat		Single Rice		Double Rice	
	2004–2008	2014–2018	2004–2008	2014–2018	2004–2008	2014–2018	2004–2008	2014–2018
NE	58.5 ± 7.1 c ^2^	59.9 ± 7.8 d	43.6 ± 2.5 e	43.3 ± 1.4 e	65.9 ± 13.5 d	61.2 ± 9.1 e	-^3^	-
NW	79.5 ± 13.6 c	84.7 ± 14.8 d	84.1 ± 14.5 c	95.1 ± 19.8 c	78.2 ± 10.4 d	77.0 ± 6.6 d	-	-
NC	292.6 ± 52.6 a	272.6 ± 47.0 a	182.3 ± 29.4 a	211.2 ± 25.3 a	98.9 ± 10.1 c	90.8 ± 5.7 c	-	-
MLYR	174.1 ± 19.5 b	184.4 ± 21.2 b	156.2 ± 8.3 b	174.0 ± 10.9 b	170.3 ± 15.7 a	168.2 ± 13.4 a	231.5 ± 15.8	245.8 ± 16.1
SE	167.5 ± 18.2 b	173.5 ± 19.9 b	-	-	122.9 ± 10.5 c	127.7 ± 11.4 b	205.3 ± 12.1	213..4 ± 10.7
SW	153.7 ± 13.0 b	151.9 ± 11.8 c	90.4 ± 8.3 d	92.3 ± 11.3 d	156.0 ± 11.0 b	141.5 ± 8.9 b	-	-
China	165.2 ± 37.5	162.4 ± 33.7	125.2 ± 35.4	140.3 ± 45.0	125.8 ± 26.2	120.4 ± 25.6	219.7 ± 12.5	231.6 ± 13.8

^1^ NC, north central; NW, northwest; MLYR, the middle and lower reaches of the Yangtze River; SE, southeast; SW, southwest. ^2^ Same letter within column means no significant difference between treatments at the level of 0.05. ^3^ “-” indicates that the crop is not planted in the area.

## Data Availability

The data presented in this study are available on request from the corresponding author.

## Supplementary Materials

The following supporting information can be downloaded at: https://www.mdpi.com/article/10.3390/plants11192507/s1, Figure S1: Annual seeding area of main crops from 2004 to 2018 in China farmlands; Figure S2: Annual seeding area of (a) maize, (b) wheat, (c) single rice and (d) double from 2004 to 2018 in China farmlands, respectively. NE, northeast; NC, north central; NW, northwest; MLYR, the middle and lower reaches of the Yangtze River; SE, southeast; SW, southwest; Figure S3: Composition of nitrogen (N) input (kg N ha-1) from 2004 to 2018 for (a) maize, (b) wheat, (c) single rice and (d) double from 2004 to 2018 in China farmlands, respectively. Figure S4. Composition of annual nitrogen (N) losses (kg N ha-1) from 2004 to 2018 for (a) maize, (b) wheat, (c) single rice and (d) double from 2004 to 2018 in China farmlands, respectively. Table S1: Summary of main arable crops and soil types at six regions of China in 2004-2018; Table S2: N content in air-dry grain and straw, ratio of grain to straw and ratio of straw returning for maize, wheat, single rice and double rice in China; Table S3: N content in air-dry grain and straw, ratio of grain to straw and ratio of straw returning for maize, wheat, single rice and double rice in China; Table S4: Livestock feeding period, daily excretion, N content and percentage for field application (based on fresh); Table S5: Nitrogen losses coefficients (%) for different components for each crop and region in China’s croplands; Table S6: Nitrogen (N) input (kg N ha-1) for maize, wheat, single rice and double rice between 2004-2008 and 2014-2018 at the region scale in China farmlands, respectively; Table S7: Nitrogen (N) output (kg N ha-1) for maize, wheat, single rice and double rice between 2004-2008 and 2014-2018 at the region scale in China farmlands, respectively; Table S8: Global warming potential (GWP) for maize, wheat, single rice and double rice between 2004-2008 and 2014-2018 at the region scale in China farmlands, respectively; Table S9: Acidification potential (AP) for maize, wheat, single rice and double rice between 2004-2008 and 2014-2018 at the region scale in China farmlands, respectively; Table S10: The water pollution level of surface runoff emissions (WPL-SR) for maize, wheat, single rice and double rice between 2004-2008 and 2014-2018 at the region scale in China farmlands, respectively; Table S11: The water pollution level of leaching emissions (WPL-LEA) for maize, wheat, single rice and double rice between 2004-2008 and 2014-2018 at the region scale in China farmlands, respectively.
