# Supplementary material for: Evaluation of Nitrogen Fertilizer Fates and Related Environmental Risks for Main Cereals in China’s Croplands from 2004 to 2018
Source: Plants (Basel). 2022 Sep 26;11(19):2507. doi: 10.3390/plants11192507 (PMC9571694; doi:10.3390/plants11192507)
Supplement: Supplementary file 1 [file plants-11-02507-s001.zip › plants-1886784-supplementary.pdf]

Evaluation of nitrogen fertilizer fates and related environmental risks for main cereals in China's croplands from 2004 to 2018

Daping Song [1], Rong Jiang [1,*], Daijia Fan [1], Guoyuan Zou [1], Lianfeng Du [1], Dan Wei [1], Xuan Guo [1], Wentian He [1,*]

[1] *Institute of Plant Nutrition, Resources and Environment, Beijing Academy of Agriculture and Forestry Sciences, 9 Shuguanghuayuan Middle Road, Haidian District, Beijing 100097, China*
∗ Corresponding authors.
E-mail addresses: wentian_he@hotmail.com (W. He), rong_jiang@outlook.com (R. Jiang)

1. Materials and Methods

1.1 Definition and estimates N emissions

1.1.1 N_2O-N emissions

N_2O is calculated based on the Intergovernmental Panel on Climate Change (IPCC) methodology [1], specific parameters of the direct emissions and indirect emissions are derived from the investigation on compiling the works of the Provincial Agricultural Greenhouse Gas Inventories in China [2]. The parameters of N_2O-N concentration per unit of economic yield based on previous studies are summarized in Table S5 [3–37]. The equation is shown as follows:

$$\mathrm{N}_{N2O} = \mathrm{N}_{input} \times \mathrm{N2O}_{emission} \quad (1)$$

where the N_{input} (kg N ha^{-1}) is the sum of chemical fertilizer N (N_{fert}), manure fertilizer N from livestock and humans (N_{man}), straw N returned to the soil (N_{straw}), N fixed via symbiotic and non-symbiotic pathways (N_{fix}), dry and wet N deposition from the atmosphere (N_{depo}) and N from irrigation water (N_{irri}). $N_2O_{emission}$ is emission factor of total N input.

1.1.2 NH_3-N emissions

NH_3-N is calculated by the chemical fertilizers and organic manures application and regional emission factors, these parameters are derived from Table S5 [3–37]. Since inputs of chemical fertilizers and organic manures are only considered in this study, the equation is shown as follows:

$$\mathrm{N}_{NH3} = \mathrm{N}_{fert} \times \mathrm{NH3}_{volatilization} + \mathrm{N}_{man} \times \mathrm{NH3}_{volatilization} \quad (2)$$

where NH_3-N is total NH_3 volatilization from cropland, (N_{fert}) is chemical fertilizer N, N_{man} is manure fertilizer N from livestock and humans NH_3 volatilization is emission factor of fertilizers volatilized as NH_3.

Table S1. Summary of main arable crops and soil types at six regions of China in 2004–2018

Region[1]	Province[2]	Maize	Wheat	Single rice	Double rice	Main soil types
Northeast (NE)	HLJ, JL, LN	HLJ, JL, LN	HLJ	HLJ, JL, LN		Black soil, cinnamon soil, meadow soil
North central (NC)	BJ, HEB, HEN, SX, SD, TJ	BJ, HEB, HEN, SX, SD, TJ	BJ, HEB, HEN, SX, SD, TJ	HEB, HEN, SD, TJ		Fluvo-aquic soil, cinnamon soil, saline-alkali soil, brown soil
Northwest (NW)	GS, NMG, NX, QH, SNX, XJ	GS, NMG, NX, SNX, XJ	GS, NMG, NX, QH, SNX, XJ	NMG, NX, SNX		Loess soil, desert soil, irrigation-silting soil, fluvo-aquic soil, chestnut soil, gray calcareous soil
The middle and lower reaches of Yangtze River (MLYR)	AH, HUB, HUN, JS, JX, SH, ZJ	AH, JS, HUB	AH, JS, HUB, SH	AH, HUB, HUN, JS, JX, SH, ZJ	AH, HUB, JX, ZJ	Fluvo-aquic soil, yellow brown soil, red soil, paddy soil
Southeast (SE)	FJ, GD, GX, HN	GX		FJ	FJ, GD, GX, HN	Red soil, yellow brown soil, purple soil, paddy soil
Southwest (SW)	CQ, GZ, SC, XZ, YN	CQ, GZ, SC, YN	CQ, GZ, SC, YN	CQ, GZ, SC, YN		Red soil, yellow brown soil, purple soil, paddy soil

[1]Six agricultural regions were grouped based on geographical locations and China's administrative divisions: NE, northeast; NC, north central; NW, northwest; MLYR, the middle and lower reaches of the Yangtze River; SE, southeast; SW, southwest.

[2]AH, Anhui; BJ, Beijing; CQ, Chongqing; FJ, Fujian; GD, Guangdong; GS, Gansu; GX, Guangxi; GZ, Guizhou; HEB, Hebei; HEN, Henan; HLJ, Heilongjiang; HN, Hainan; HUB, Hubei; HUN, Hunan; JL, Jilin; JS, Jiangsu; JX, Jiangxi; LN, Liaoning; NMG, Inner Mongolia; NX, Ningxia; QH, Qinghai; SC, Sichuan; SD, Shandong; SH, Shanghai; SNX, Shaanxi; SX, Shanxi; TJ, Tianjin; XJ, Xinjiang; XZ, Tibet; YN, Yunnan; ZJ, Zhejiang.

Table S2. N content in air-dry grain and straw, ratio of grain to straw and ratio of straw returning for maize, wheat, single rice and double rice in China [38–55].

Crop	Region	N content in grain (%)	N content in straw (%)	Grian /straw	Ratio of straw returning (%)
Maize	NE	2.25	0.89	0.90	25
	NW	2.25	0.89	0.77	50
	NC	2.25	0.89	0.81	80
	MLYR	2.25	0.89	0.77	80
	SE	2.25	0.89	0.79	50
	SW	2.25	0.89	0.78	30
Wheat	NE	2.12	0.57	0.64	85
	NW	2.12	0.57	0.91	82
	NC	2.12	0.57	0.75	90
	MLYR	2.12	0.57	0.72	88
	SW	2.12	0.57	0.67	85
Single rice	NE	1.30	0.82	1.10	45
	NW	1.30	0.82	0.97	55
	NC	1.30	0.82	1.00	70
	MLYR	1.30	0.82	0.93	72
	SE	1.30	0.82	1.08	72
	SW	1.30	0.82	1.05	50
Double rice	MLYR	1.30	0.82	0.93	72
	SE	1.30	0.82	1.08	72

[1]NC, north central; NW, northwest; MLYR, the middle and lower reaches of the Yangtze River; SE, southeast; SW, southwest.

Table S3. N input parameters of non-fixation N, deposition N and irrigation N for maize, wheat, single rice and double rice [51–54, 56–58].

Crop	Region[1]	Non-fixation ($kg\ ha^{-1}$)	Deposition ($kg\ ha^{-1}$)	Irrigation ($kg\ ha^{-1}$)
Maize	NE	15.0	10.8	3.5
	NW	15.0	7.2	5.0
	NC	15.0	18.2	6.9
	MLYR	15.0	26.6	5.2
	SE	15.0	26.2	3.4
	SW	15.0	15.3	3.1
Wheat	NE	15.0	9.4	7.9
	NW	15.0	9.4	7.9
	NC	15.0	12.9	9.9
	MLYR	15.0	14.5	5.8
	SW	15.0	14.5	5.8
Single rice	NE	33.0	9.4	7.7
	NW	33.0	9.4	7.7
	NC	33.0	12.9	9.9
	MLYR	33.0	14.5	5.8
	SE	33.0	14.5	5.8
	SW	33.0	14.5	5.8
Double rice	MLYR	44.0	19.3	7.7
	SE	44.0	19.3	7.7

[1]NC, north central; NW, northwest; MLYR, the middle and lower reaches of the Yangtze River; SE, southeast; SW, southwest.

Table S4. Livestock feeding period, daily excretion, N content and percentage for field application (based on fresh) [2,57].

Livestock	Feeding phase	Population Structure (%)	Feeding Period (d)	Daily excretion/urine and N content[1]			Rate to field (%)	
				Excretion (kg d)	Urine (kg d)	Content (%)	Urine (%)	2000s–2010s
Human		100	365	0.31	1.60	1.16	0.53	33
Dairy	Adult cattle	53	365	37.5	18.8	0.43	0.87	30
	Young dairy cattle	37	365	21.4	10.7	0.43	0.87	30
	Calf	10	180	5.80	2.9	0.43	0.87	30
Beef	Heifers for slaughter	73	365	18.1	9.1	0.38	0.51	30
	Fattening cattle	16	270	12.1	6.0	0.38	0.51	30
	Calf	11	120	4.00	2.0	0.38	0.51	30
Pig	Breeding pigs	11	365	5.50	3.0	0.55	0.17	65
	Fattening pigs	56	180	4.00	3.0	0.55	0.17	65
	Piglets	37	90	1.50	3.0	0.55	0.17	65
Sheep/Goat	Adult sheep/goat	70	365	2.60	-	0.89	-	33
	Lambs	30	180	1.40	-	0.89	-	33
Poultry		100	210	0.13	-	0.94	-	45
Horse		100	365	10.0	5.0	0.42	0.69	44
Donkey		100	365	10.0	5.0	0.45	0.71	44
Mule		100	365	10.0	5.0	0.33	0.60	44

[1] The N contents in daily excretion and urine were evaluated together based on parameters availability.

Table S5. Nitrogen losses coefficients (%) for different components for each crop and region in China's croplands [3–37].

Crop	Region[1]	N_2O emission		NO emission		N_2 emission		NH_3 volatilization		N runoff		N leaching	
		2004–2008	2014–2018	2004–2008	2014–2018	2004–2008	2014–2018	2004–2008	2014–2018	2004–2008	2014–2018	2004–2008	2014–2018
Maize	NE	0.65	0.65	0.52	0.52	9.70	9.71	6.69	6.67	0.28	0.29	2.69	2.7
	NW	0.68	0.69	0.54	0.55	10.14	10.31	6.47	6.44	0.27	0.28	5.97	6.05
	NC	1.66	1.61	1.33	1.29	24.94	24.14	11.74	11.81	0.83	0.80	20.67	21.12
	MLYR	0.89	0.91	0.71	0.73	13.31	13.71	7.60	7.58	1.05	1.09	11.27	11.38
	SE	0.87	0.88	0.70	0.71	13.10	13.26	7.61	7.60	1.06	1.08	10.7	10.94
	SW	0.85	0.84	0.68	0.67	12.69	12.64	7.63	7.64	0.95	0.94	8.57	8.72
Wheat	NE	0.32	0.32	0.26	0.26	4.80	4.80	8.41	8.41	0.28	0.28	4.71	4.7
	NW	0.36	0.37	0.29	0.30	5.36	5.56	7.73	7.67	0.27	0.29	8.27	8.95
	NC	0.46	0.47	0.37	0.38	6.91	7.06	7.53	7.49	0.83	0.88	19.33	19.51
	MLYR	1.39	1.48	1.11	1.18	20.78	22.17	12.83	12.84	1.05	1.11	7.26	8.03
	SW	1.06	1.07	0.85	0.85	15.87	15.99	12.76	12.76	0.95	0.97	4.61	4.68
Single rice	NE	0.25	0.25	0.20	0.20	3.78	3.80	11.12	11.21	1.24	1.22	2.61	2.62
	NW	0.26	0.26	0.20	0.20	3.84	3.83	10.94	10.96	1.33	1.32	2.65	2.64
	NC	0.27	0.27	0.22	0.21	4.10	3.98	10.76	10.82	1.53	1.44	2.83	2.75
	MLYR	0.83	0.83	0.67	0.66	12.48	12.40	19.87	19.87	3.90	3.88	2.67	2.66
	SE	0.74	0.76	0.59	0.61	11.03	11.47	18.44	18.42	2.37	2.38	2.61	2.61
	SW	1.00	0.88	0.80	0.70	14.95	13.20	18.27	18.33	2.43	2.40	2.63	2.62
Double rice	MLYR	0.72	0.73	0.58	0.58	10.81	10.95	19.82	19.83	3.38	3.43	2.97	2.98
	SE	0.58	0.61	0.46	0.48	8.65	9.09	18.62	18.58	2.35	2.35	2.97	2.97

[1] NC, north central; NW, northwest; MLYR, the middle and lower reaches of the Yangtze River; SE, southeast; SW, southwest.

Table S6. Nitrogen (N) input (kg N ha^{-1}) for maize, wheat, single rice and double rice between 2004–2008 and 2014–2018 at the region scale in China farmlands, respectively.

Region[1]	Maize		Wheat		Single rice		Double rice	
	2004–2008	2014–2018	2004–2008	2014–2018	2004–2008	2014–2018	2004–2008	2014–2018
NE	284.9±61.2c	292.0±36.7b	200.2±15.3c	198.7±8.8c	343.1±92.7c	317.2±59.4e	-[2]	-
NW	379.1±35.0ab	397.3±36.6a	346.6±77.8b	378.4±102.7b	406.9±68.9b	401.0±43.7bc	-	-
NC	411.1±72.6a	395.9±62.3a	429.8±83.7a	454.3±64.7a	501.9±65.8a	466.3±37.3a	-	-
MLYR	388.5±27.6ab	397.3±34.9a	351.9±36.8b	371.7±46.8b	421.3±57.7b	417.3±49.2b	604.3±36.4	637.5±37.4
SE	380.3±17.2ab	388.4±32.5a	-	-	343.5±6.28c	349.5±5.86de	610.4±49.2	625.9±43.6
SW	362.8±44.9b	360.3±37.6a	250.4±42.9c	254.0±58.2c	389.2±45.2bc	370.8±36.8cd	-	-
China	367.8±64.4	371.9±57.0	346.1±95.1	366.6±105.7	414.0±79.6	398.8±63.6	607.0±42.2	632.3±40.2

[1] NC, north central; NW, northwest; MLYR, the middle and lower reaches of the Yangtze River; SE, southeast; SW, southwest.

[2] "-" indicates that the crop is not planted in the area.

Table S7. Nitrogen (N) output (kg N ha^{-1}) for maize, wheat, single rice and double rice between 2004–2008 and 2014–2018 at the region scale in China farmlands, respectively.

Region[1]	Maize		Wheat		Single rice		Double rice	
	2004–2008	2014–2018	2004–2008	2014–2018	2004–2008	2014–2018	2004–2008	2014–2018
NE	241.8±23.3d	260.5±28.4e	146.1±20.1d	173.0±6.3c	255.3±21.7e	269.3±25.4d	-[2]	-
NW	298.8±43.5c	336.4±45.6c	217.6±34.1c	244.0±44.2b	273.2±23.5de	286.0±13.8cd	-	-
NC	385.9±45.2a	393.3±38.2a	337.8±42.3a	388.5±26.8a	300.5±28.0bc	318.6±24.8b	-	-
MLYR	339.7±25.3b	379.0±30.1b	301.4±23.6b	331.5±29.3b	363.7±22.4a	382.5±23.2a	560.0±20.4	615.6±22.1
SE	312.2±23.0c	320.2±23.6d	-	-	304.4±9.7cd	319.5±8.9c	509.8±38.1	535.0±39.8
SW	315.0±22.9bc	335.7±27.6cd	169.4±27.0d	177.0±28.7c	352.4±25.5ab	354.4±19.4b	-	-
China	351.2±65.2	373.8±62.2	255.3±62.4	285.8±69.7	320.4±33.3	234.3±33.8	537.5±33.8	579.5±41.2

[1] NC, north central; NW, northwest; MLYR, the middle and lower reaches of the Yangtze River; SE, southeast; SW, southwest.

[2] "-" indicates that the crop is not planted in the area.

Table S8. Global warming potential (GWP) for maize, wheat, single rice and double rice between 2004–2008 and 2014–2018 at the region scale in China farmlands, respectively.

Region[1]	Maize		Wheat		Single rice		Double rice	
	2004–2008	2014–2018	2004–2008	2014–2018	2004–2008	2014–2018	2004–2008	2014–2018
NE	139.2±28.4b	228.4±58.9b	4.9±0.24c	2.0±0.60c	25.4±10.1c	34.6±25.2b	-[2]	-
NW	65.8±45.0b	112.2±77.2bc	23.7±14.5c	29.4±15.4c	3.1 ±0.43c	3.2±0.82b	-	-
NC	313.7±192.4a	373.9±199.4a	110.3±76.5b	128.9±82.1b	7.5±6.79c	7.6±8.07b	-	-
MLYR	52.4±12.3b	79.6±19.5c	181.4±86.3a	257.4±112.4a	122.3±83.1a	124.9±88.1a	204.7±121.6	204.9±126.2
SE	54.7±5.5b	63.6±4.8c	-	-	71.6±4.5b	23.7±1.18b	147.3±93.1	140.7±97.8
SW	88.8±40.1b	107.3±46.5bc	52.5±32.7bc	35.7±20.4c	139.1±77.4a	112.0±61.5a	-	-
China	145.3±85.9	191.8±107.4	83.1±55.2	101.2±76.2	72.7±61.5	67.7±56.1	179.2±106.2	176.4±102.6

[1] NC, north central; NW, northwest; MLYR, the middle and lower reaches of the Yangtze River; SE, southeast; SW, southwest.

[2] "-" indicates that the crop is not planted in the area.

Table S9. Acidification potential (AP) for maize, wheat, single rice and double rice between 2004–2008 and 2014–2018 at the region scale in China farmlands, respectively.

Region[1]	Maize		Wheat		Single rice		Double rice	
	2004–2008	2014–2018	2004–2008	2014–2018	2004–2008	2014–2018	2004–2008	2014–2018
NE	8.73±1.78b	14.3±3.68a	0.77±0.04b	0.32±0.10b	6.80±2.70cd	9.28±6.76bc	-[2]	-
NW	3.82±2.68c	6.37±4.60b	3.11±1.90b	3.69±2.46b	0.80±0.12d	0.82±0.20d	-	-
NC	13.4±10.1a	16.6±12.5a	10.9±10.46a	12.4±12.35a	1.79±1.75d	1.87±2.10d	-	-
MLYR	2.72±0.64c	4.00±0.98b	10.2±7.53a	13.56±10.06a	17.71±12.1a	18.20±12.9a	34.2±27.8	33.7±29.5
SE	2.89±0.29c	3.32±0.25b	-	-	10.89±0.68bc	3.46±0.25cd	28.9±17.4	26.2±17.3
SW	4.85±2.19bc	5.90±3.18b	3.84±3.31b	2.59±2.65b	15.46±8.54ab	14.15±7.74ab	-	-
China	7.11±6.92	9.70±8.74	6.72±7.65	7.71±9.47	10.31±10.47	10.24±9.93	31.8±23.8	30.4±25.1

[1] NC, north central; NW, northwest; MLYR, the middle and lower reaches of the Yangtze River; SE, southeast; SW, southwest.

[2] "-" indicates that the crop is not planted in the area.

Table S10. The water pollution level of surface runoff emissions (WPL-SR) for maize, wheat, single rice and double rice between 2004–2008 and 2014–2018 at the region scale in China farmlands, respectively.

Region[1]	Maize		Wheat		Single rice		Double rice	
	2004–2008	2014–2018	2004–2008	2014–2018	2004–2008	2014–2018	2004–2008	2014–2018
NE	0.0034±0.0018b	0.0050±0.0018b	0.0001±0.0000b	0.0000±0.0001b	0.0058±0.0008b	0.0070±0.0023b	-[2]	-
NW	0.0028±0.0019b	0.0043±0.0028b	0.0024±0.0026b	0.0018±0.0015b	0.0128±0.0173ab	0.0119±0.0158ab	-	-
NC	0.0379±0.0206a	0.0428±0.0244a	0.0363±0.0250a	0.0383±0.0276a	0.0065±0.0019b	0.0069±0.0035b	-	-
MLYR	0.0021±0.0007b	0.0030±0.0009b	0.0035±0.0042b	0.0079±0.0068b	0.0242±0.0237a	0.0235±0.0241a	0.0138±0.0079	0.0135±0.0089
SE	0.0007±0.0001b	0.0008±0.0001b	0.0031±0.0046b	0.0003±0.0002b	0.0038±0.0004b	0.0010±0.0000b	0.0081±0.0020	0.0072±0.0024
SW	0.0014±0.0003b	0.0017±0.0003b	-	-	0.0049±0.0017b	0.0043±0.0012b	-	-
China	0.0120±0.0192	0.0141±0.0218	0.0123±0.0204	0.0130±0.0220	0.0125±0.0169	0.0121±0.0168	0.0113±0.0066	0.0107±0.0075

[1] NC, north central; NW, northwest; MLYR, the middle and lower reaches of the Yangtze River; SE, southeast; SW, southwest.

[2] "-" indicates that the crop is not planted in the area.

Table S11. The water pollution level of leaching emissions (WPL-LEA) for maize, wheat, single rice and double rice between 2004–2008 and 2014–2018 at the region scale in China farmlands, respectively.

Region[1]	Maize		Wheat		Single rice		Double rice	
	2004–2008	2014–2018	2004–2008	2014–2018	2004–2008	2014–2018	2004–2008	2014–2018
NE	0.0045±0.0021b	0.0066±0.0019b	0.0004±0.0000b	0.0002±0.0001b	0.0017±0.0003b	0.0020±0.0005ab	-[2]	-
NW	0.0023±0.0011b	0.0038±0.0018b	0.0065±0.0055b	0.0059±0.0035b	0.0006±0.0005b	0.0006±0.0005c	-	-
NC	0.0680±0.0313a	0.0772±0.0405a	0.0578±0.0306a	0.0727±0.0428a	0.0008±0.0004b	0.0010±0.0007bc	-	-
MLYR	0.0076±0.0024b	0.0116±0.0034b	0.0072±0.0050b	0.0105±0.0078b	0.0030±0.0022a	0.0029±0.0022a	0.0007±0.0004	0.0006±0.0004
SE	0.0032±0.0003b	0.0 038±0.0003b			0.0008±0.0001b	0.0003±0.0001c	0.0006±0.0001	0.0005±0.0002
SW	0.0066±0.0025b	0.0074±0.0021b	0.0007±0.0003b	0.0003±0.0002b	0.0013±0.0007b	0.0011±0.0006bc	-	-
China	0.0221±0.0255	0.0259±0.0272	0.0199±0.0164	0.0245±0.0181	0.0017±0.0015	0.0014±0.0011	0.0006±0.0003	0.0006±0.0003

[1] NC, north central; NW, northwest; MLYR, the middle and lower reaches of the Yangtze River; SE, southeast; SW, southwest.

[2] "-" indicates that the crop is not planted in the area.

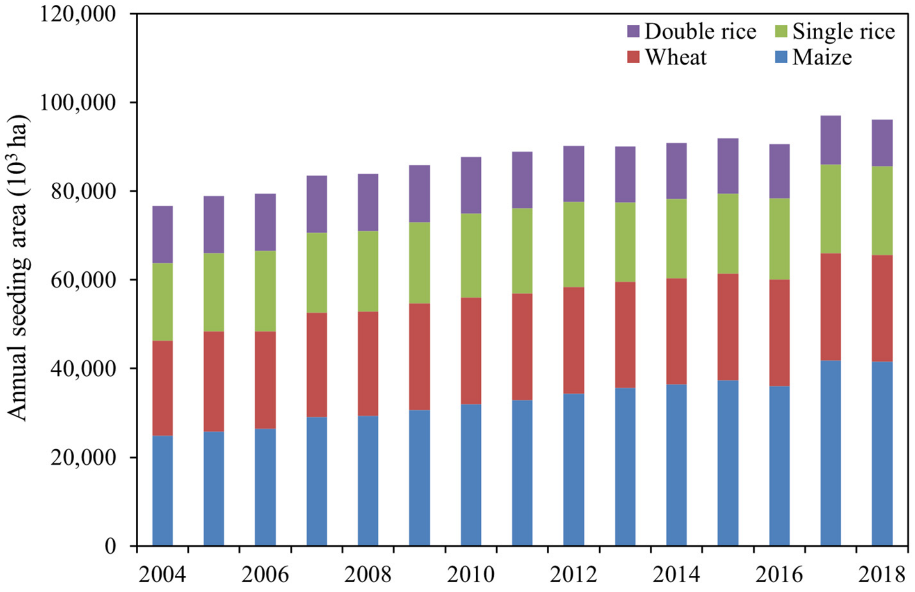

Figure. S1. Annual seeding area of main crops from 2004 to 2018 in China farmlands.

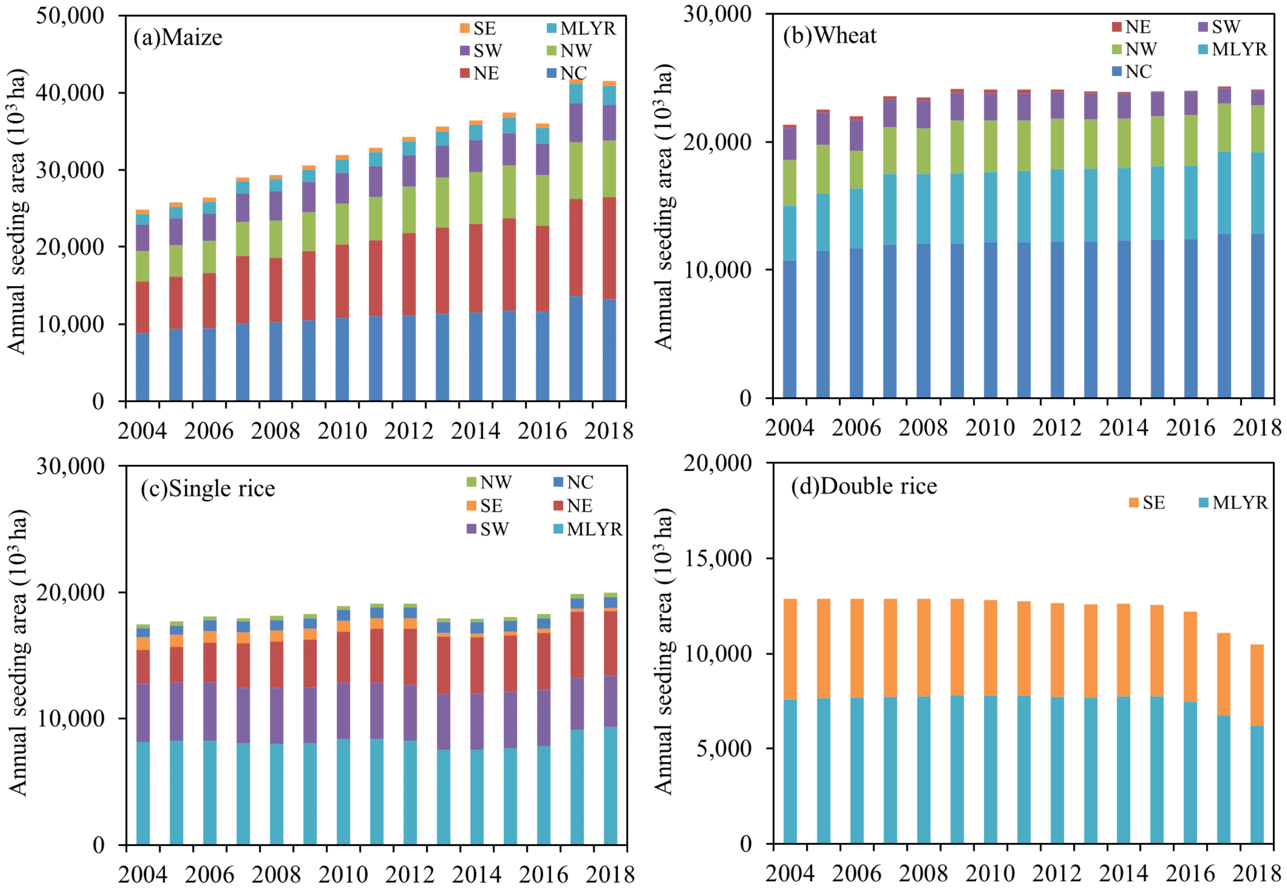

Figure S2. Annual seeding area of (a) maize, (b) wheat, (c) single rice and (d) double from 2004 to 2018 in China farmlands, respectively. NE, northeast; NC, north central; NW, northwest; MLYR, the middle and lower reaches of the Yangtze River; SE, southeast; SW, southwest.

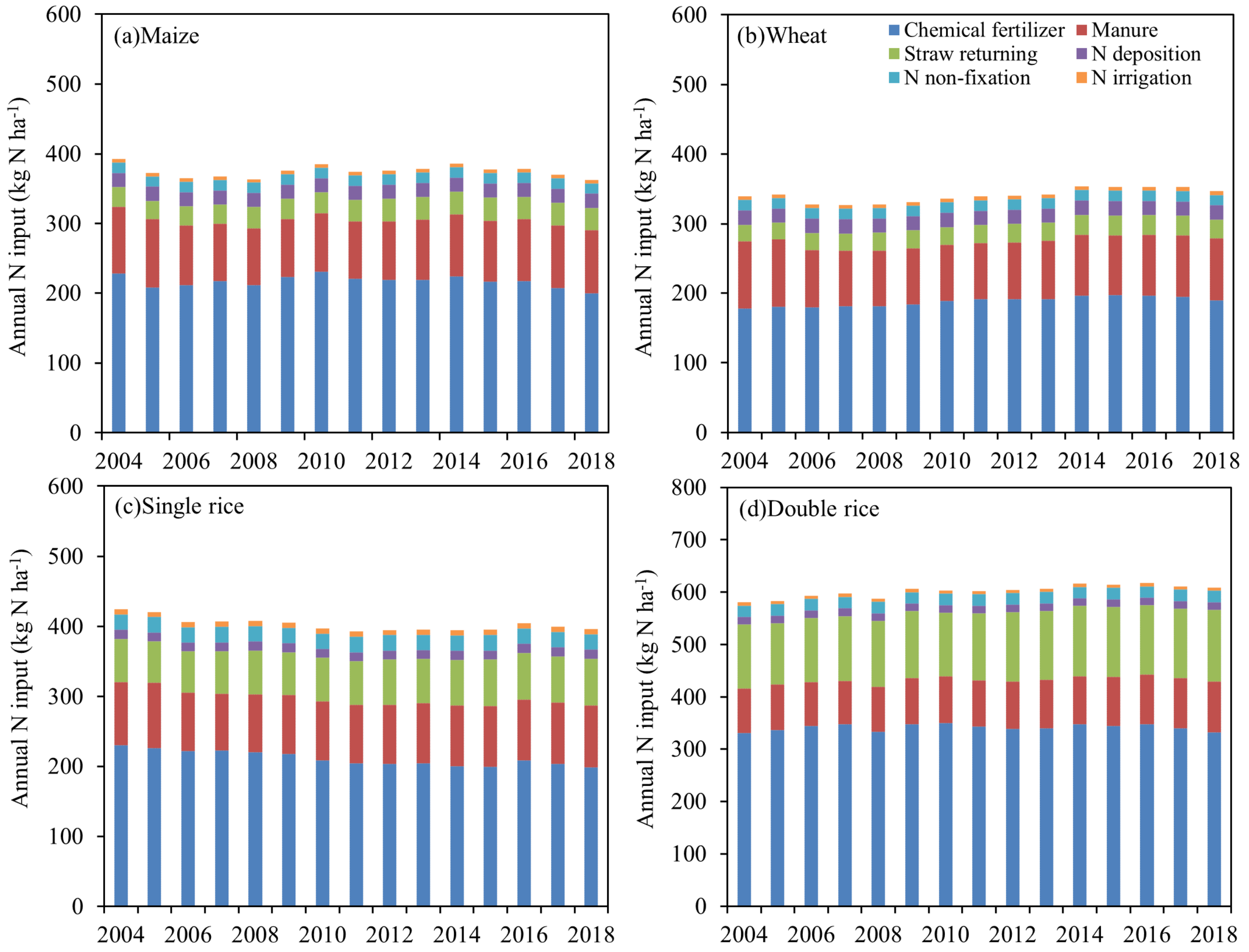

Figure S3. Composition of nitrogen (N) input (kg N ha^{-1}) from 2004 to 2018 for (a) maize, (b) wheat, (c) single rice and (d) double from 2004 to 2018 in China farmlands, respectively.

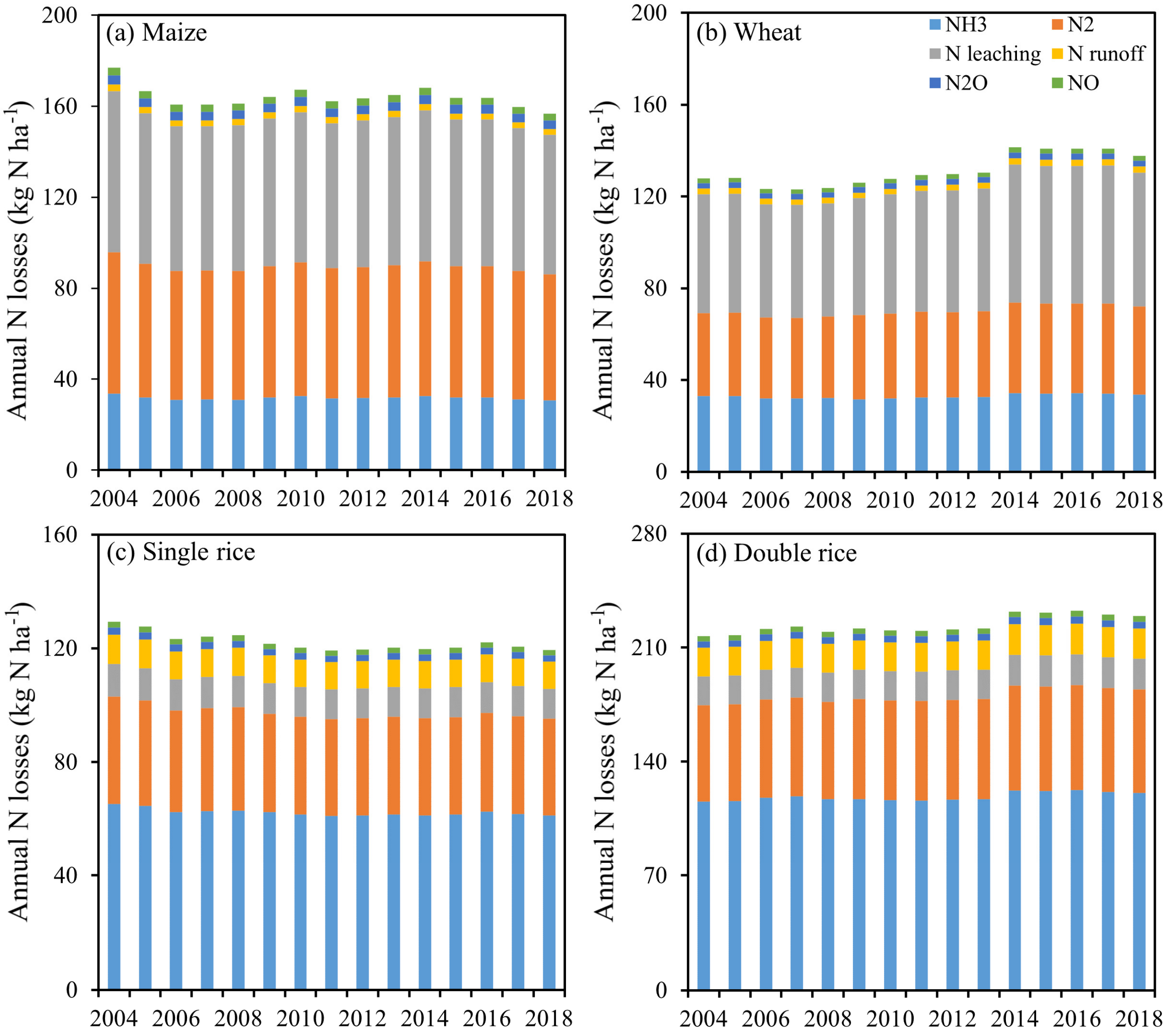

Figure S4. Composition of annual nitrogen (N) losses (kg N ha^{-1}) from 2004 to 2018 for (a) maize, (b) wheat, (c) single rice and (d) double from 2004 to 2018 in China farmlands, respectively.

Reference

1. IPCC. Climate Change 2014: Synthesis Report. Contribution of Working Groups I, II and III to the Fifth Assessment Report of the Intergovernmental Panel on Climate Change [Core Writing Team, R.K. Pachauri and L.A. Meyer (eds.)]. IPCC, Geneva, Switzerland, 2014, pp. 87.
2. Hu, Y.; Chen, H.; Duan, L. Investigation on compiling work of provincial agricultural greenhouse gas Inventories. Henan Sci. 2016, 34 (5), 692-697, (in Chinese with English abstract).
3. Zhang, X.; Wu, Y.; Liu, X.; Reis, S.; Jin, J.; Dragosits, U.; Van, D.M.; Clarisse, L.; Whitburn, S.; Coheur, P.F. Ammonia emissions may be substantially underestimated in China. Environ. Sci. Technol. 2017, 51(21).
4. Gui, J.; Li, Z.; Deng, X.; Yang, J.; Chen, D.; Li, W. An analysis of spatiotemporal patterns in Chinese agricultural productivity between 2004 and 2014. Ecol. Indicat. 2018, 105, 591–660.
5. He, W.T.; Jiang, R.; He, P.; Yang, J.Y.; Zhou, W.; Ma, J.C.; Liu, Y.X. Estimating soil nitrogen balance at regional scale in China's croplands from 1984 to 2014. Agr. Syst. 2018, 167:125–135.
6. Ren, T. Z.; Liu, H. B.; Fan, X. B.; Zhou, G. Y.; Liu, S. Emission coefficient manual of agricultural non-point source pollution in China. Beijing: China Agriculture Press, 2018. (in Chinese with English abstract).
7. Ding, W.C.; He, P.; Zhang, J.J.; Liu, Y.X.; Xu, X.P.; Ullaha, S.; Cui, Z.L.; Zhou, W. Optimizing rates and sources of nutrient input to mitigate nitrogen, phosphorus, and carbon losses from rice paddies. J. Clean. Prod. 2020, 256, 120603.
8. Sun, C. ; Chen, L. ; Zhai, L.M. ; Liu, H.B. ; Wang, K. ; Jiao, C. ; Shen, Z.Y. National assessment of nitrogen fertilizers fate and related environmental impacts of multiple pathways in China. J. Clean. Prod. 2020, 277(11), 123519.
9. Cui, Z.L.; Zhang, H.Y.; Chen, X.P.; Zhang, C.J.; Ma, W.Q.; Huang, C.D.; Zhang, W.F.; Mi, G.H.; Miao, Y.X.; Li, X.L.; Gao, Q.; Yang, J.C.; Wang, Z.H.; Ye, Y.L.; Guo, S.W.; Lu, J.W.; Huang, J.L.; Lv, S.H.; Sun, Y.X.; Liu, Y.Y.; Peng, X.L.; Ren, J.; Li, S.Q.; Deng, X.P.; Shi, X.J.; Zhang, Q.; Yang, Z.P.; Tang, L.; Wei, C.Z.; Jia, L.L.; Zhang, J.W.; He, M.R.; Tong, Y.N.; Tang, Q.Y.; Zhong, X.H.; Liu, Z.H.; Cao, N.; Kou, C.L.; Ying, H.; Yin, Y.L.; Jiao, X.Q.; Zhang, Q.S.; Fan, M.S.; Jiang, R.F.; Zhang, F.S.; Dou, Z.X. Pursuing sustainable productivity with millions of smallholder farmers. Nature 2018, 555, 363–366.
10. Ding, W.C.; He, P.; Zhang, J.J.; Liu, Y.X.; Xu, X.P.; Ullah, S.; Cui, Z.L.; Zhou, W. Optimizing rates and sources of nutrient input to mitigate nitrogen, phosphorus, and carbon losses from rice paddies. J. Clean. Prod. 2020, 256.
11. Ju, X.T.; Lu, X.; Gao, Z.L.; Chen, X.P.; Su, F, Kogge, M.; Römheld, V.; Christie, P.; Zhang, F.S. Processes and factors controlling N2O production in an intensively managed low carbon calcareous soil under sub-humid monsoon conditions. Environ Pollut. 2011, 159(4):1007–1016.
12. Sang, M.M.; Fan, H.; Jiang, S.S.; Jiang J.Y. Nitrogen Loss Through different ways in cropland under conventional fertilization: an in-situ study of summer maize season in the middle and

lower reaches of the yangtze river. Environ. Sci. 2015, 36(9): 3358–3364. (in Chinese with English abstract)

13. Gao, D.C.; Zhang, L.; Liu, Q.; Rong X.M.; Zhang, Y.P.; Tian, C. Effects of different fertilization modes on soil nitrogen runoff in dryland field. J Soil Water Conserv. 2014, 28(3): 209–213. (in Chinese with English abstract)
14. Guo, Q.P.; Liang, S.; Kan, Y.j.; Huang, B.y.; Lei, Z.x.; Li, Y.s.; Du, J.J. Yield effect of sweet corn and characteristics of farmland nitrogen and phosphorus runoff losses under reduced fertilizer rate. Chin. Agric. Sci. Bull. 2021, 37(9): 71–78. (in Chinese with English abstract)
15. Hong, L.; Li, R.H. Characteristics of nitrogen and phosphorus losses in surface runoff from farmland in a typital irrigation district in southern China. Geogr Res-aust. 2011, 30(1): 115–124. (in Chinese with English abstract)
16. Pei, H.W.; Shen, Y.J.; Liu, C.M. Nitrogen and water cycling of typical cropland in the North China Plain. Chin. J. Appl. Ecol. 2015, 26(1): 283–296. (in Chinese with English abstract)
17. Lin, D.X.; Fan, X.H.; Hu, F.; Zhao, H.T.; Luo, J.F. Ammonia volatilization and nitrogen utilization efficiency in response to urea application in rice fields of the Taihu lake region, China. Pedosphere, 2007, 17(5): 639–645.
18. Ju, X.T.; Xing, G.X.; Chen, X.P.; Zhang, S.L.; Zhang, L.J.; Liu, X.J.; Liu, X.J.; Cui, Z.L.; Yin, B.; Christie, P.; Zhu, Z.L.; Zhang, F.S. Reducing environmental risk by improving N management in intensive Chinese agricultural systems (Proceedings of the National Academy of Sciences of the United States of America, 2009,106(9): 3041–3046.
19. Wang, Z.H.; Liu, X.J.; Ju, X.T.; Zhang, F.S. In situ determination of ammonia volatilization from wheat-maize rotation system field in north China. Acta Ecologica Sinica. 2002, 22(3): 359-365.
20. Li, Z.X.; Dong, S.T.; Wang, K.J.; Zhang, J.W.; Liu, P.; Wang, Q.C.; Liu, C.X. In situ study on influence of different fertilization strategies for summer maize on soil nitrogen leaching and volatilization. Plant Nutr. Fert. Sci. 2007, 13(6): 998–1005. (in Chinese with English abstract)
21. Lv, J.L.; Wang X.F.; Li T.K.; Kou, C.L. Ammonia emission characteristics and emission coefficients of wheat and corn rotation cropland under different fertilization methods in lime concretion black soil. Chinese Journal of Eco-Agriculture, 2020, 28(12): 1869-1879. (in Chinese with English abstract)
22. Li, G.T.; Li, B.G.; Chen, D.L. Ammonia volatilization from large field planted with winter wheat and summer maize. Act Agriculture Boreali Sinica. 2002, 17(1): 76–81.
23. Zhan, R.F.; Chen, X.P.; Zhang, F.S. Nitrogen cycling and balance in winter-wheat summer-maize rotation system in northern China plain. Acta Pedologica Sinica. 2009, 46(4): 684–697.
24. Hu M.M.; Dong, W.X.; Wang, W.Y.; Gaudel, G.; Mosongo, P.; Hu, C.S. The effects of deep application of nitrogen fertilization on ammonia volatilization in a winter wheat/summer maize rotation system in the North China Plain. Chin. J. Eco-Agric. 2020, 28(12): 1880–1889. (in Chinese with English abstract)
25. Su, F.; Ding, X.Q.; Gao, Z.L.; Huang, B.X.; Chen, X.P.; Zhang, F.S.; Kogge, M.; Volker, R. Ammonia volatiiization from nitrogen fertilization of winter wheat-summer nlIaize rotation

system in the North China Plain. China Environ. Sci. 2007, 27(3): 409–413. (in Chinese with English abstract)
26. Shen, S.Z.; Zhang, K.Q.; Wang, F. The assessment method of nitrate nitrogen leaching and ammonia volatilization of winter wheat-summer maize rotation in North China plain based on RZWQM model. J. Eco. Rur. Environ. 2018, 34(9): 769–775. (in Chinese with English abstract)
27. Xie, Y.; Rong, X.M.; Liu, Q.; Zhang, Y.P. Effects of controlled release nitrogen fertilizer application on nitrogen dynamics and loss of surface runoff in spring maize fieid. J. Soil Water Conserv. 2016, 30(1): 14–25. (in Chinese with English abstract)
28. Ma, Y.L.; Ji, Y.z.; Li, X.; Zhang, L.; Ju, X.t.; Zhang L.J. Effects of N fertilization rates on the nh, volatilization and no emissions from the wheat-maize rotation system in North China plain. Ecol Environ. 2012, 21(2): 225–230. (in Chinese with English abstract)
29. Zhang, M.; Zhao, M.; Tian, Y.H.; Yin, B.; Zhu, Z.L. Study on N leaching and runoff under integrated high yield and high efficiency practices in paddy fields of Taihu lake region. Soils, 2018, 50(1): 35–42. (in Chinese with English abstract)
30. Zhu, Z.L.; Fan, X.H.; Sun, Y.H.; Wang, D.J. Nitrogen cycle and its environmental effects in paddy soil of Taihu lake. Crop Res. 2004, (4): 187–191. (in Chinese with English abstract)
31. Fan, H.X.; Xu, L.; Zhao, X.; Hu, Y.F. Study on nitrogen loss in rice-wheat rotation farmland in Taihu basin. Ecol Environ. 2015, 24(2): 255–262. (in Chinese with English abstract)
32. Xia, Y.Q.; Wang, S.Q.; Sun, P.F.; Chen, X.Q.; Shen, J.L.; Wang, H.; Xiao, Z.H.; Li, X.M.; Yang, G.; Yan, X.Y. Ammonia emission patterns of typical planting systems in the middle and lower reaches of the Yangtze River and key technologies for ammonia emission reduction. Chin. J. Eco-Agric. 2021, 29(12): 1981−1989. (in Chinese with English abstract)
33. Feng, X.J.; Zheng, Z.C.; Li, T.G. Characteristics of runoff and nitrogen loss in sloping cropland of purple soil during corn growing season. J. Soil Water Conserv. 2017, 31(1): 43–54. (in Chinese with English abstract)
34. Wang, A.; Dai, D.C.; Ma, X.Z.; Mou, Q.; Yu Y.Q.; Lv, W.Q. Effects of rice-crab culture on nitrogen leaching in rice fields in the north of China. Journal of Zhejiang University (Agric. & Life Sci.), 2019, 45(3): 332–342. (in Chinese with English abstract)
35. Ma, R.Y.; Yu, K.; Xiao, S.Q.; Liu, S.W.; Ciais, P.; Zou, J.W. Data-driven estimates of fertilizer-induced soil NH_3, NO and N_2O emissions from croplands in China and their climate change impacts. Global Change Biol. 2021, 28, 1008–1022.
36. Chen, T.; Oenema, O.; Li, J.Z.; Misselbrook, T.; Dong, W.X.; Qin, S.P; Yuan, H.J.; Li, X.X.; Hu, C.S. Seasonal variations in N_2 and N_2O emissions from a wheat–maize cropping system. Biol. Fert. Soils. 2019, 55:539–551.
37. Zhang, Y.K.; Wang, R.; Pan, Z.L.; Liu, Y.; Zheng, X.H.; Ju, X.T.; Zhang, C.; Klausa, B.B.; Huang, B.X. Fertilizer nitrogen loss via N_2 emission from calcareous soil following basal urea application of winter wheat. Atmos. Oceanic Sic. Lett. 2019, 12(2) : 91–97.
38. Lu, F.; Wang, X.K.; Han, B.; Ouyang, Z.Y.; Duan, X.N.; Zheng, H.; Miao, H. Soil carbon sequestrations by nitrogen fertilizer application, straw return and no-tillage in China's cropland. Global Change Biol. 2010, 15(2):281–305.

39. Yao, Z.L.; Zhao, L.X.; Tian, Y.S.; Meng, H.B. Utilization status and medium and long-term forecast of crop straw resource in Heilongjiang Province. Trans. Chin. Soc. Agric. Eng. 2009,25(11): 288–292. (in Chinese with English abstract)
40. Cui, M.M.; Jiang, L.L.; Yan, T.W. Potential evaluation and market assessment on crop straw resource utilization based on resourced density. J. China Agric. Univ. 2016, 21(6): 117–131. (in Chinese with English abstract)
41. Wang, F.; Shi, Z.L.; Li, X.; Chang, Z.Z.; Liu, L.L.; Sun, R.H. Discussion on concept, design idea and technology model of regionaltotal straw utilization. Chinese J. Agric. Res. & Regio. Plain. 2016, 37(5): 8–12. (in Chinese with English abstract)
42. Bao, J.C.; Yu, J.H.; Feng, Z.; Chen, B.H.; Lei, C.; Yang, J. Situation of distribution and utilization of crop straw resources in seven western provinces, China. Chin. J. Appl. Ecol. 2014, 25(1): 181–187. (in Chinese with English abstract)
43. Liu, H.Y.; Wu J.S.; Zhou, J.G.; Xiao, H.A.; Zhou, P. Regional differences of inputs of organic matter and chemical fertilizer in south central China. Chin. J. Appl. Ecol. 2015, 26 (9): 2721–2727. (in Chinese with English abstract)
44. Zhang Y.M.; Sun, H.Y.; Li, H.J.; Liu, X.J.; Hu, C.S.; Liu, K.T.; Cui, Y.X.; Zhang, M.Y. Nutrient cycling and balance in farmland ecosystem in Bohai Lowland Plain. Chinese Journal of Eco-Agriculture, Aug. 2016, 24(8): 1035–1048. (in Chinese with English abstract)
45. Fu, Y.H.; Liu, L.M.; Yuan, C.C. Comprehensive evaluation for nitrogen footprint and grey water footprint of agricultural land use system. Trans. Chin. Soc. Agric. Eng. 2016, 32(Supp.1): 312–319. (in Chinese with English abstract)
46. Bao, X.; Watanabe, M.; Wang, Q.; Hayashi, S.; Liu, J. Nitrogen budgets of agricultural fields of the Changjiang River basin from 1980 to 1990. Sci. Total Environ. 2006, 363, 136–148.
47. Bi, Y.; Gao, C.; Wang, Y.; Li, B. Y. Estimation of straw resources in China. Trans. CSAE. 2009, 25(12), 211–217, (in Chinese with English abstract).
48. China National Agricultural Technology Extension Service. Organic Fertilizer resources in China. China Agriculture Press, Beijing. 1999a. (in Chinese)
49. China National Agricultural Technology Extension Service. Data Collection for Organic Fertilizer Nutrients in China. China Agriculture Press, Beijing. 1999b. (in Chinese)
50. Gao, L.; Ma, L.; Zhang, W.; Ma, W.; Zhang, F. Estimation of nutrient resource quantity of crop straw and its utilization situation in China. Trans. CSAE. 2009, 25(7), 173–179. (in Chinese with English abstract)
51. Li, S.; Jin, J.. Characteristics of nutrient input/output and nutrient balance in different regions of China. Sci. Agric. Sin. 2011, 44 (20), 4207–4229. (in Chinese with English abstract)
52. Cui, S.H.; Shi, Y.L.; Groffman, P.M.; Schlesinger, W.H.; Zhu, Y.G. Centennial-scale analysis of the creation and fate of reactive nitrogen in China (1910-2010). Proc. Natl. Acad. Sci. USA 2013, 110 (6), 2052-2057.
53. Wang, X.L.; Feng, A.P.; Wang, Q.; Wu, C.Q.; Liu, Z.; Ma, Z.S.; Wei, X.F. Spatial variability of the nutrient balance and related NPSP risk analysis for agro-ecosystems in China in 2010. Agric. Ecosyst. Environ. 2014, 193, 42–52.

54. Gu, B.J.; Ju, X.H.; Chang, J.; Ge, Y.; Vitousek, P.M. Integrated reactive nitrogen budgets and future trends in China. Proc. Natl. Acad. Sci. USA 2015, 112 (28), 792–8797.
55. Liu, X.Y.; Li, S.T. Temporal and spatial distribution characteristics of crop straw nutrient resources and returning to farmland in China. Trans. CSAE. 2017, 33(21), 1–19, (in Chinese with English abstract).
56. Liu, X.Y.; Li, S.T. Temporal and spatial distribution of nutrient resource from livestock and poultry feces and its returning to cropland. Trans. CSAE. 2018, 34(4), 1–14, (in Chinese with English abstract).
57. Chuan L.M.; He P.; Zhao T.K.; Xu X.P.; Zhou W.; Zhen H.G. Nitrogen cycling and balance for wheat in China. Chin. J. Appl. Ecol. 2015, 26(1), 76–86. (in Chinese with English abstract)
58. Xu, P.X.; Han, L.L.; He, J.Z.; Lu, F.; Zhang, L.M. Research advance on molecular ecology of asymbiotic nitrogen fixation microbes. Chin. J. Appl. Ecol. 2017, 28(10), 3440–3450.
